# HIV-1 activates oxidative phosphorylation in infected CD4 T cells in a human tonsil explant model

**DOI:** 10.3389/fimmu.2023.1172938

**Published:** 2023-05-30

**Authors:** Tracey L. Freeman, Connie Zhao, Nadine Schrode, Trinisia Fortune, Sanjana Shroff, Benjamin Tweel, Kristin G. Beaumont, Talia H. Swartz

**Affiliations:** ^1^ Medical Scientist Training Program, University of Pittsburgh-Carnegie Mellon University, Pittsburgh, PA, United States; ^2^ Department of Medicine, Icahn School of Medicine at Mount Sinai, New York, NY, United States; ^3^ Department of Genetics and Genomic Sciences, Icahn School of Medicine at Mount Sinai, New York, NY, United States; ^4^ Division of Infectious Diseases, Department of Medicine, Icahn School of Medicine at Mount Sinai, New York, NY, United States; ^5^ Department of Otolaryngology, Icahn School of Medicine at Mount Sinai, New York, NY, United States

**Keywords:** HIV-1, NLRP3 inflammasome, oxidative phoshorylation, bystander cells, CD4 T cell, macrophage, metabolomics, single cell RNA sequencing (scRNA)

## Abstract

**Introduction:**

Human immunodeficiency virus type 1 (HIV-1) causes a chronic, incurable infection leading to immune activation and chronic inflammation in people with HIV-1 (PWH), even with virologic suppression on antiretroviral therapy (ART). The role of lymphoid structures as reservoirs for viral latency and immune activation has been implicated in chronic inflammation mechanisms. Still, the specific transcriptomic changes induced by HIV-1 infection in different cell types within lymphoid tissue remain unexplored.

**Methods:**

In this study, we utilized human tonsil explants from healthy human donors and infected them with HIV-1 *ex vivo*. We performed single-cell RNA sequencing (scRNA-seq) to analyze the cell types represented in the tissue and to investigate the impact of infection on gene expression profiles and inflammatory signaling pathways.

**Results:**

Our analysis revealed that infected CD4^+^ T cells exhibited upregulation of genes associated with oxidative phosphorylation. Furthermore, macrophages exposed to the virus but uninfected showed increased expression of genes associated with the NLRP3 inflammasome pathway.

**Discussion:**

These findings provide valuable insights into the specific transcriptomic changes induced by HIV-1 infection in different cell types within lymphoid tissue. The activation of oxidative phosphorylation in infected CD4^+^ T cells and the proinflammatory response in macrophages may contribute to the chronic inflammation observed in PWH despite ART. Understanding these mechanisms is crucial for developing targeted therapeutic strategies to eradicate HIV-1 infection in PWH.

## Introduction

Human immunodeficiency virus type 1 (HIV-1) leads to a chronic, incurable infection that progresses to acquired immunodeficiency syndrome (AIDS) when unsuppressed by lifelong antiretroviral therapy (ART). Despite the efficacy of ART in suppressing viral load, people with HIV (PWH) experience significantly higher rates of age-associated comorbidities, including cardiovascular disease, neurocognitive decline, malignancies, and decreased life expectancy (1–4). Many of these comorbidities have been attributed to the chronic, dysfunctional, hyperactive inflammatory state associated with HIV-1 infection (1–7).

The molecular mechanisms underlying this abnormal inflammatory state are multifactorial and largely unknown. HIV-1 infection leads to plasmacytoid dendritic cell (pDC) decline, and overexpression of proinflammatory cytokines, such as interferon α (IFNα), may skew T-cell differentiation in favor of activated effector T cells (8, 9). HIV-1-induced intestinal barrier defects allow microbial translocation in amounts that exceed the host immune defense mechanisms. This leads to persistently high extracellular lipopolysaccharide (LPS) and bacterial DNA that likely drive chronic immune activation (10, 11). Impaired suppression of co-infections such as cytomegalovirus (CMV) contributes significantly to persistent T cell activation (12). Elevated circulating levels of LPS, as well as extracellular nucleotides released by inflamed or apoptotic cells, may lead to activation of purinergic receptors, Toll-like receptors (TLRs), and the downstream NACHT, LRR, and PYD domain-containing protein 3 (NLRP3) inflammasome signaling axis (13). An emerging body of literature has implicated the critical role of the NLRP3 inflammasome in mediating inflammatory signaling in HIV-1 infection and inflammation and inflammatory cell death, known as pyroptosis (14–37).

The tonsils, a form of mucosa-associated lymphoid tissue (MALT), are a primary location of the latent HIV-1 reservoir and are likely mediators of HIV-associated acute and chronic inflammation (38, 39). Unlike the peripheral blood, tonsils act as sequestered viral reservoirs during the clinical latency period with active, progressive viral replication, storage, and persistence, even in the presence of ART (40–42). A defining feature of the tonsils is an intricate and well-defined spatial arrangement of diverse immune cell types. Follicular regions rich in B cells are surrounded by extrafollicular regions composed primarily of CD4+ T cells. There are extensive epithelial, endothelial, and myeloid cell networks (43). This precise architecture facilitates the propagation and amplification of immune cascades between individual cells and cell types at border zones. The prominent role of cytoarchitecture in mediating tonsillar immune responses highlights the importance of studying these pathways in their native spatial arrangements (43–48).

Human tonsil explant models preserve the native cellular repertoire of lymphoid tissue and crucial cell-cell interactions (49, 50). HIV-1 predominantly infects activated CD4^+^ T cells in single-cell culture, leading to direct killing *via* apoptosis (6, 7, 51). Tonsil tissue explants can support productive HIV-1 infection without exogenous activation, as PBMCs require. When challenged with recall antigens, tonsil tissue blocks can produce a specific antibody response modulated by HIV-1 infection in a pattern similar to that observed *in vivo* (52). In tonsil explant cultures, HIV-1 has been shown to induce the secretion of proinflammatory cytokines. These effects, however, are not seen in PBMCs (39, 42, 53, 54) or single cell culture of dissociated tonsil tissue (unpublished data). CD4+ T cells in the tonsils have been implicated in triggering inflammatory cascades in neighboring immune cells, rendering them more susceptible to abortive infection and pyroptosis, a form of inflammatory cell death (53, 55). These phenotypic variations may reflect the crucial role of tonsillar architecture in the HIV-1 immune response.

Identifying distinct and essential subpopulations of cells within the diverse cellular makeup of human tissue has remained a technical challenge. RNA-sequencing of bulk tissue provides an overall average transcriptomic profile of the sample but masks contributions from individual cells and subpopulations, especially in heterogeneous samples. With the advent of single-cell sequencing technologies, it has become possible to characterize subpopulations of cells and their critical contributions to understanding phenomena such as cellular mechanisms of infection and immune response. This increased resolution characterizes the transcriptome of each cell in a population, permitting the identification of subpopulations that are both genetically similar and distinct. Our human tonsil explant model employs a single-cell RNA-sequencing (scRNA-seq) approach to characterize the cell type-specific transcriptional effects of HIV-1 infection and associated inflammation. Here, we use scRNA-seq in an *ex vivo* human tonsil explant model to define the immune cell populations hosting HIV-1 infection and investigate the roles of specific immune cell subtypes in mediating HIV-associated inflammatory and metabolic pathogenesis in native lymphoid tissue.

## Results

### Infection, processing, and RNA sequencing of tonsillar cells

**Figure 1:**
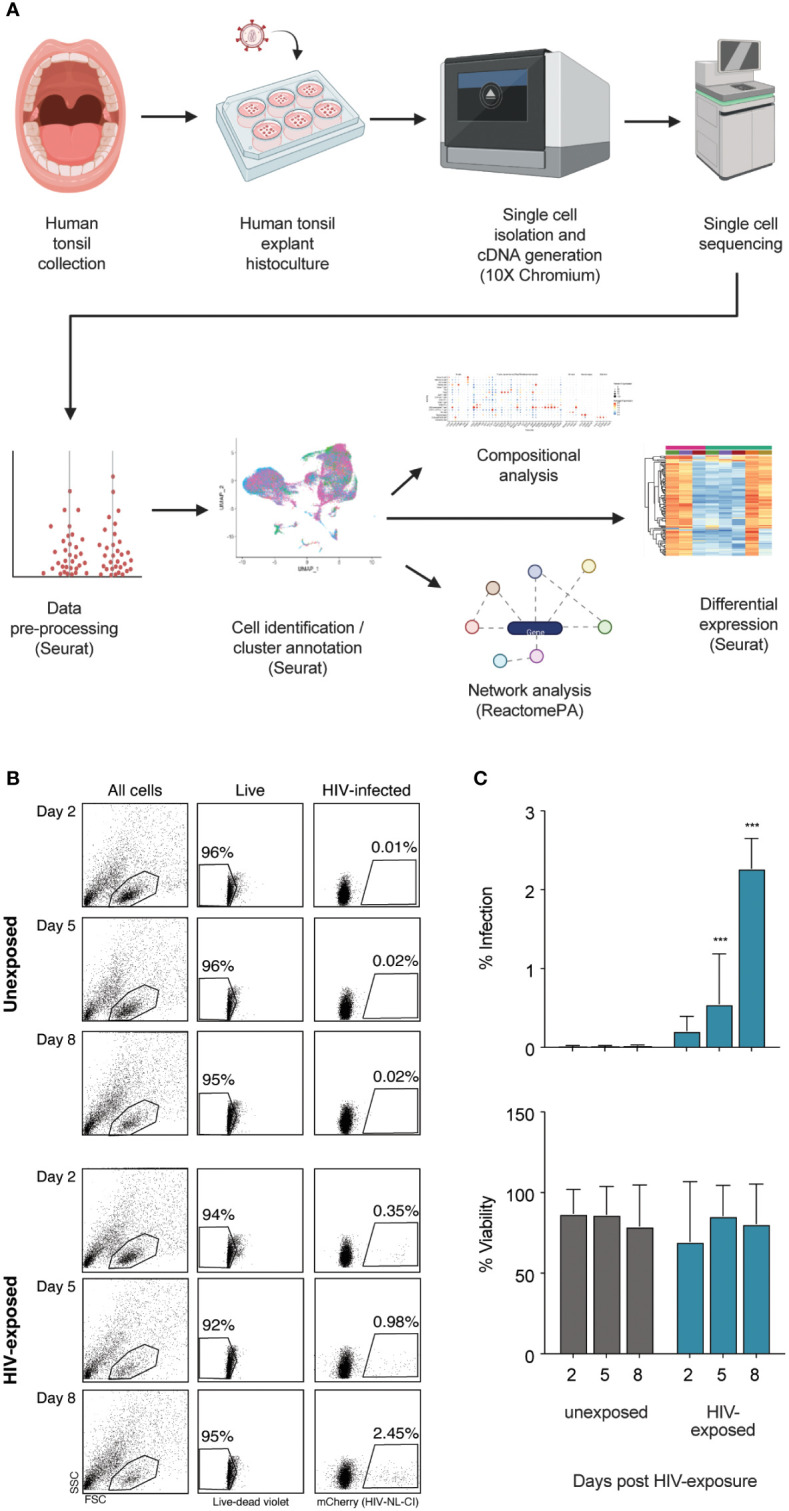
Infection of human tonsil explants by X4-tropic HIV-1_NL-CI_. The scheme shows the collection of human tonsil explants, infection with HIV-1_NL-CI_, single-cell isolation, and cDNA generation using 10X Chromium, single-cell RNA sequencing, data pre-processing, cell identification and cluster annotation using Seurat, and compositional, network, and differential analysis using Seurat and ReactomePA (Created with Biorender.com) **(A)**. Human tonsil explants were cultured on collagen rafts and infected with HIV-1_NL-CI_ or vehicle media. Supernatants were collected on days 2, 5, and 8 post-infection. Sloughed-off cells and media were separated by centrifugation, and a full media change was performed on each indicated time cells were collected. Cells were subjected to LIVE/DEAD staining and flow cytometry to evaluate viability. Suspension cells in supernatants were collected on day 8 for single-cell dissociation and processing for single-cell sequencing **(B)**. Flow cytometry results are quantified as percent infection and percent viability in tonsils unexposed or exposed to HIV-1. Mean values ± standard errors of the means from four donors **(C)**. *, P ≤ 0.05; **, P ≤ 0.01; ***, P ≤ 0.001.

A broad overview of human tonsil preparation, exposure to HIV-1_NL-CI_, a CXCR4-tropic virus including a fluorescent mCherry reporter, sequencing, and analysis is shown in **Figure 1A**. The viability and infection rate of the cells were quantified by flow cytometry through LIVE/DEAD stain and mCherry expression, respectively. Eight days after HIV exposure, 95% of cells were determined to be alive, and 2.45% of live cells were productively HIV-infected (**Figures 1B, C**).

**Figure 2:**
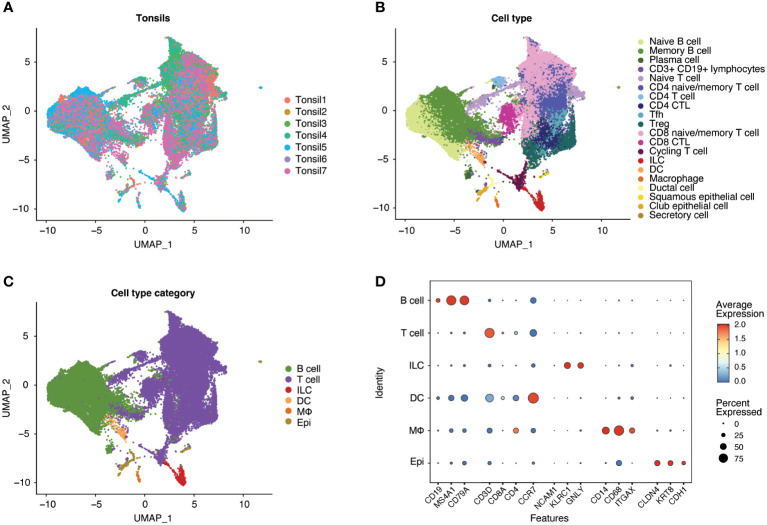
Human tonsil explants contain CD4^+^ T, CD8^+^ T, and B cells with smaller populations of innate lymphoid cells (ILC), dendritic cells (DC), macrophages, and epithelial cells. Single-cell datasets from human tonsil explants of 7 donors (Tonsil 1-7) were merged and integrated. Uniform Manifold Approximation and Projection (UMAP) plots of the resultant populations of 47686 cells are color-coded by tonsil donor **(A)**, cell type based on marker expression in clusters, and **(B)** summarized into cell type categories **(C)**. As illustrated by dot plot **(D)**, cell type identification and cluster annotation were based on known immune and non-immune cell marker gene expression patterns. Dot size is proportional to the percentage of cells within a compartment. The dot color indicates the average expression across the cluster (red = high, blue = low).

Following scRNA-seq of exposed and unexposed tonsil samples from seven donors, the integrated data (**Figure 2A**) was analyzed through Louvain clustering and cell type and broader cell type category annotation was performed (**Figures 2B, C**) based on the expression of canonical markers (**Figure 2D**). The seven tonsil samples comprised largely lymphocytes, including T cells (CD4^+^ and CD8^+^) and B cells with small populations of innate lymphoid cells (ILC), macrophages, dendritic cells (DC), and epithelial cells (**Figure 2C**), consistent with expected cellular yields from a tonsil explant model system. Analysis of cell types is shown in **Supplemental Figure 1**.

### Establishing infection based on HIV-1 alignment and characterization of infected cell clusters

**Figure 3:**
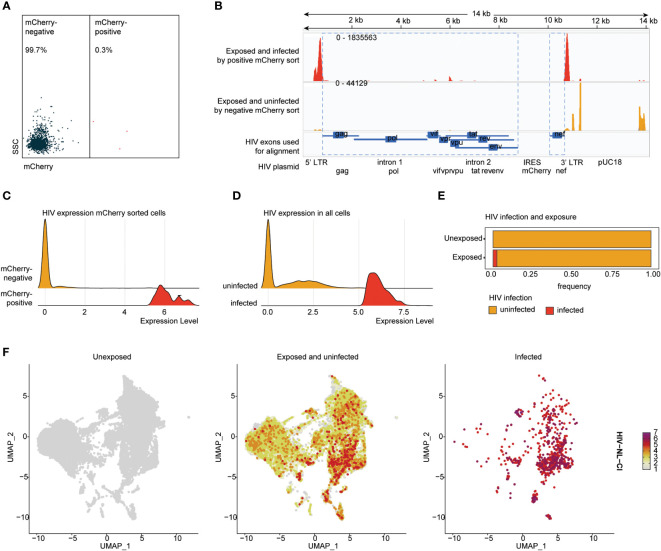
HIV-1_NL-CI_ transcript expression aligns with HIV-1 productive infection in human tonsils. Human tonsil explants exposed to HIV-1_NL-CI_ were subject to flow sorting to separate mCherry negative and mCherry positive cells **(A)**. Sequencing reads were aligned to the genome of HIV-1_NL-CI_ with exposed and infected by positive mCherry sort in red and exposed and uninfected by mCherry sort in yellow. The map of the HIV-1_NL-CI_ genome (56) shows where sequencing reads were aligned. Using a joint GRCh38-2020-A Human reference and a custom HIV reference whose regions with known features are marked as exonic (in blue), the pipeline grouped and de-duplicated reads mapped to the transcriptome using 10X cellular barcodes and UMIs (Unique Molecular Identifiers) **(B)**. Ridge plots illustrate the distribution of detected HIV transcript in mCherry-positive and negative sorted cells **(C)** and in all cells, split by cell groups defined as infected and uninfected based on a 2.45% cutoff **(D)**. Exposed tonsils demonstrate 2.45% infection **(E)**. Split UMAP showing total cells by HIV groups, unexposed, exposed uninfected, and infected **(F)**.

To understand the association betweenthe detection of HIV-1 transcripts and HIV productive infection, HIV-exposed cells were sorted by mCherry fluorescence to detect productively infected cells (**Figure 3A**). Sorted cells were analyzed by scRNA-seq, including alignment to the HIV-1 NLCI reference (61) to characterize the read alignment profile of productively infected cells, as shown in **Figure 3B**. HIV expression in mCherry-sorted cells showed a clear separation of infected and uninfected cells (**Figure 3C**). This distribution corresponded well with previous results indicating that 2.45% of exposed cells exhibited productive infection (**Figures 1B, C** **)**. Based on this, a cutoff of the top 2.45% of HIV-exposed cells was established, such that in subsequent analyses, those with levels of HIV transcript above that threshold were considered infected. The total cells, unexposed and exposed to HIV, are indicated in **Figures 3D, E**, showing 2.45% of infected cells in the HIV-exposed cells. **Figure 3F** shows the distribution of unexposed, exposed uninfected, and infected cells after dimensionality reduction (UMAP plots). **Supplemental Figure 2** shows the UMAP of unexposed, exposed uninfected, and infected cells (**SI Figure 2A**), each tonsil donor by the distribution of cell exposure and infection (**SI Figure 2B**), and ridge plots of HIV transcripts by tonsil donor (**SI Figure 2C**).

### Profiling the immune cell landscape during HIV-1 infection

**Figure 4:**
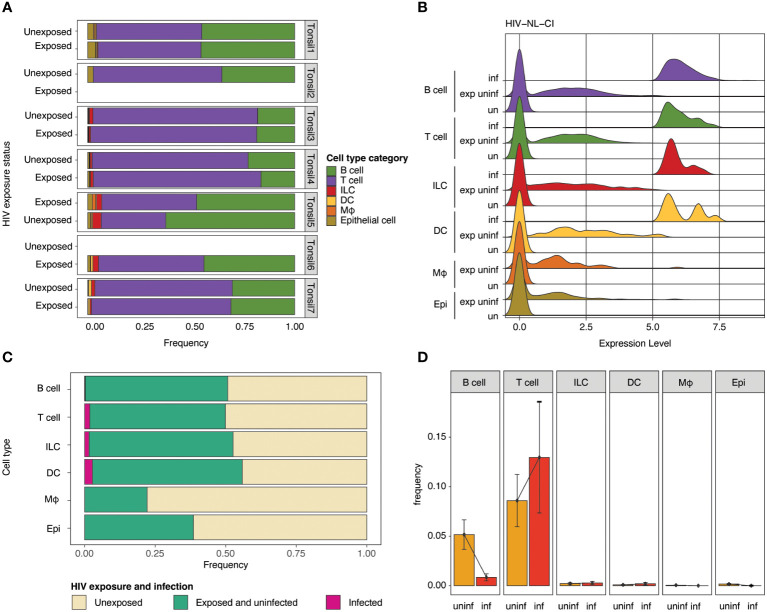
Impact of HIV-1_NL-CI_ exposure on cell category. Stacked bar plots illustrate relative frequencies of cell categories in the integrated dataset, stratified by tonsil donor and HIV-1 exposure **(A)**. Ridge plots show the distribution of HIV-1 transcript expression in each cell type by HIV-1 exposure group: unexposed, exposed uninfected, and infected **(B)**. Stacked bar plots show each cell type’s relative abundance of infected cells **(C)**. Bar plots show the frequency of HIV-1 infection by cell type. Error bars denote the standard error of the mean **(D)**. “exp uninf” = exposed uninfected, “un”/”uninf” = uninfected, “inf” = infected.

The impact of HIV-1 exposure on the distribution of cell category (**Figure 4A**) and cell type (**Supplemental Figure 3A**) was determined within each tonsil to evaluate the immune cell landscape. While considerable heterogeneity was noted among the seven samples, no notable differences in cell category or cell type distribution were noted between exposed and unexposed donors. Samples of exposed and unexposed tonsils were available for Donor 1, 3, 4, 5, and 7, unexposed tonsil samples were available for Donor 1, 2, 3, 4, 5, and 7, and exposed tonsil samples were available for Donor 1, 3, 4, 5, 6, and 7. Ridge plots of HIV-1 transcript expression level are shown by cell category in **Figure 4B** and cell types in **Supplemental Figure 3B**. High levels of HIV-1 transcript were noted in the B cell, T cell, ILC, and DC compartments (**Figure 4B**). In contrast, no infected cells were observed in macrophages or epithelial populations. For the subsequent analysis, groups were compared based on three categories, unexposed, exposed uninfected, and infected. The normalized relative abundance of infected cells in cell type categories is shown in stacked box plots in **Figure 4C** (and cell category and type in **Supplemental Table 1**). T cells and DCs comprised the largest relative groups of infected cells, while a lesser part of ILCs and a negligible percentage of B cells exhibited infection. Finally, as above, no macrophages or epithelial cells were productively infected. HIV infection frequency is shown in each cell category (**Figure 4D**) and cell type (**Supplemental Figure 3C**), with the highest frequency of infection in T cells. While HIV expression was noted in B cells, **Figures 4C, D** show a significantly lower number of B cells among the infected cells compared to uninfected cells. **Figure 4C** also indicates that each cell category had relatively comparable viability between unexposed and exposed cells in B cells, T cells (CD4 and CD8), ILCs, and DCs. In contrast, epithelial cells showed a modest reduction, and macrophages had the greatest reduction in representation in exposed compared to unexposed cells.

### Differential gene expression in infected cells reveals activated oxidative phosphorylation

**Figure 5:**
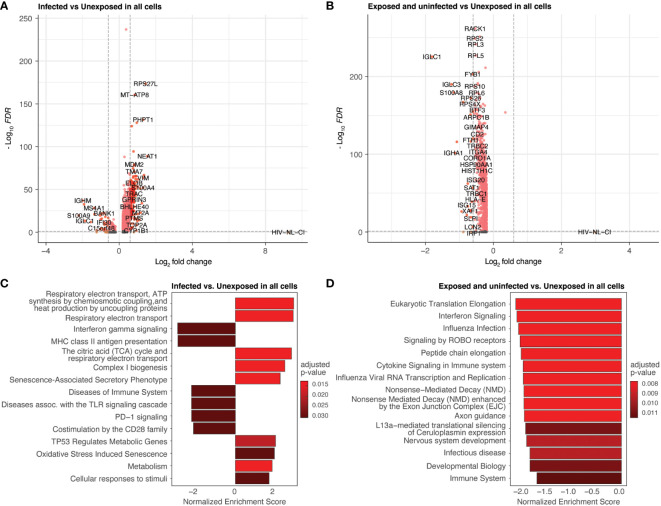
Differential expression by HIV-1_NL-CI_ infection and exposure. Volcano plots of genes differentially expressed in infected cells compared to unexposed cells **(A)** and in exposed uninfected cells compared to unexposed cells **(B)**. Colors denote significance and fold change cutoffs: red = absolute log2(fold change) ≥ 0.6 and adjusted p-value < 0.05, pink = absolute log2(fold change) < 0.6 and adjusted p-value <0.05, orange = absolute log2(fold change) ≥ 0.6 and adjusted p-value >0.05, grey = absolute log2(fold change) < 0.6 and adjusted p-value >0.05. Bar plots illustrate the top 15 Reactome database gene sets enriched in infected cells vs. unexposed cells **(C)** and exposed uninfected cells vs. unexposed cells **(D)**. Gene sets with adjusted p-value <0.05 were considered significant. A positive Normalized Enrichment Score (NES) value indicates enrichment.

Differential gene expression was performed by comparing all infected cells to unexposed cells (**Figures 5A, C**; **Supplemental Table 2**) and all exposed but uninfected cells to unexposed cells (**Figures 5B, D**; **Supplemental Table 3**). Volcano plots (**Figures 5A, B**) show the fold change of gene expression between infected cells (5A) as well as exposed uninfected cells (5B) compared to unexposed cells, with the y-axis indicating statistical significance. The corresponding bar plots show the top 15 Reactome (reactome.org) gene sets enriched in the infected (**Figure 5C**) or exposed uninfected (**Figure 5D**) compared to unexposed cells, ranked by Normalized Enrichment Score (NES), with positive values indicating enrichment and negative values depletion. Among the top gene sets enriched in infected cells were several related to cellular respiration, including 1) respiratory electron transport, ATP synthesis by chemiosmotic coupling and heat production by uncoupling proteins (R-HSA-163200), 2) Respiratory electron transport (R-HSA-6111050), and 3) The citric acid (TCA) cycle and respiratory electron transport (R-HSA-1428517). R-HSA-16320 is related explicitly to uncoupling proteins during oxidative phosphorylation. Uncoupling proteins are mitochondrial transporters that dissipate the proton gradient across the inner mitochondrial membrane. R-HSA-6111050 is more broadly associated with respiratory electron transport, the process by which electrons are transferred along the electron transport chain in the inner mitochondrial membrane to generate a proton gradient and ATP. R-HSA-1428517 is related to both the citric acid cycle and respiratory electron transport. The citric acid cycle occurs in the mitochondrial matrix and generates high-energy electron carriers such as NADH and FADH2, which are used in the electron transport chain to generate ATP.

These gene sets were not prominently enriched in the exposed uninfected cells, where more disparate pathways were noted (**Figure 5D**). These included cytokine signaling in the immune system and Signaling by ROBO receptors gene sets related to cellular processes involved in responding to environmental stimuli and maintaining cellular homeostasis. Another major group of gene sets represented included several protein translation and interferon signaling gene sets.

**Figure 6:**
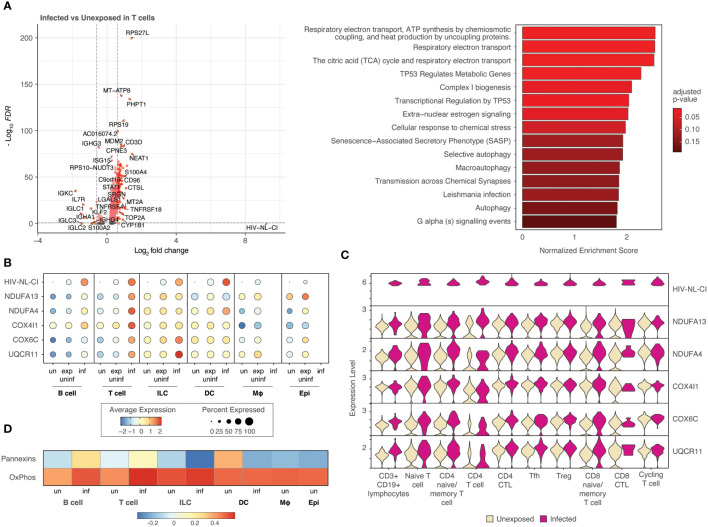
T cells infected with HIV-1 demonstrate increased oxidative phosphorylation pathways. Volcano plots of genes differentially expressed, and bar plot illustrating the top 15 Reactome database gene sets enriched in infected T cells vs. unexposed T cells. Colors denote significance and fold change cutoffs: red = absolute log2(fold change) ≥ 0.6 and adjusted p-value < 0.05, pink = absolute log2(fold change) < 0.6 and adjusted p-value <0.05, orange = absolute log2(fold change) ≥ 0.6 and adjusted p-value >0.05, grey = absolute log2(fold change) < 0.6 and adjusted p-value >0.05. Gene sets with adjusted p-value <0.05 were considered significant. A positive Normalized Enrichment Score (NES) value indicates enrichment **(A)**. Dot plots demonstrate the expression of 5 oxidative phosphorylation genes in each of the five cell categories. Dot size is proportional to the percentage of cells within a group. The dot color indicates the average expression across the group (red = high, blue = low) **(B)**. Violin plots of the five oxidative phosphorylation genes are shown by expression in ten T cell populations **(C)**. A heatmap demonstrates the relative expression of gene signatures of pannexin genes and genes involved in oxidative phosphorylation in each of the cell types **(D)**. “exp uninf”, exposed uninfected; “un”/”uninf”, uninfected; “inf”, infected.

To further probe the impact of HIV infection on differential gene expression, cell categories were explored individually. **Figure 6** (and **Supplemental Table 4**) demonstrates the differential gene expression in infected T cells compared to unexposed T cells. A volcano plot (**Figure 6A**) and bar plot (**Figure 6B**) demonstrate the same cellular respiration gene sets mentioned above 1) Respiratory electron transport, ATP synthesis by chemiosmotic coupling, and heat production by uncoupling proteins (R-HSA-163200), 2) Respiratory electron transport (R-HSA-6111050), and 3) The citric acid (TCA) cycle and respiratory electron transport (R-HSA-1428517). This similarity to the parallel comparison in all cells likely stems from the high abundance of T cells among infected cells. This result further supports the notion that HIV-1 infected T cells display increased activity in oxidative phosphorylation pathways relative to uninfected T cells and exposed uninfected T cells. Of note, this trend was not observed in exposed and uninfected T cells relative to unexposed T cells (**Supplemental Figures 4A, B**; **Supplemental Table 5**), suggesting that the upregulation of oxidative phosphorylation pathways is specific to the infected state of T cells.

Five representative oxidative phosphorylation genes (*NDUFA13, NDUFA4, COX4I1, COX6C, UQCR11)* were noted to be expressed most highly in infected T cells among all cell categories with intermediate expression in exposed uninfected T cells and the lowest expression in unexposed T cells (**Figure 6B**). Subsets of T cells exhibited increased expression of each of these genes in infected cells compared to uninfected, as shown in the violin plots in **Figure 6C** and **Supplemental Figure 4C**. This more highly resolved cell type analysis of the genes listed demonstrates that infected naïve or memory T cells, cytotoxic CD4 T cells (CD4 CTL), and T follicular helper cells (Tfh) exhibit the highest comparative expression of this gene group, followed by regulatory T cells (Tregs).

Further analysis revealed that a gene signature comprising pannexin-1 and pannexin-2 (“Pannexins”) was highly upregulated in infected T cells, following a similar trend as oxidative phosphorylation gene signatures (“OxPhos”), as shown in **Figure 6D**. These findings suggest that pannexins may act as a conduit to mediate ATP flux from infected T cells. This may represent communication by ATP release between infected cells and nearby cells.

### Macrophages exposed to HIV-1 upregulate the NLRP3 inflammasome

**Figure 7:**
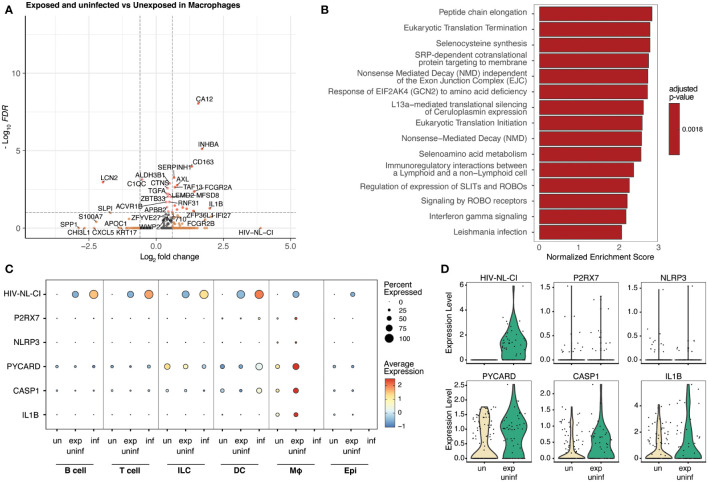
Macrophages exposed to HIV but not infected demonstrate inflammatory signaling increases, including the NLRP3 inflammasome. Volcano plots of genes differentially expressed **(A)**, and bar plot illustrating 15 representative top Reactome database gene sets enriched in exposed uninfected macrophages vs. unexposed macrophages **(B)**. Colors denote significance and fold change cutoffs: red = absolute log2(fold change) ≥ 0.6 and adjusted p-value < 0.05, pink = absolute log2(fold change) < 0.6 and adjusted p-value <0.05, orange = absolute log2(fold change) ≥ 0.6 and adjusted p-value >0.05, grey = absolute log2(fold change) < 0.6 and adjusted p-value >0.05 **(A)**. Gene sets with adjusted p-value <0.05 were considered significant. A positive Normalized Enrichment Score (NES) value indicates enrichment **(B)**. Dot plots demonstrate the expression of five NLRP3 inflammasome genes in five cell categories. Dot size is proportional to the percentage of cells within a group. The dot color indicates the average expression across the group (red = high, blue = low) **(C)**. Violin plots of the NLRP3 inflammasome gene expression are shown in macrophages **(D)**. “exp uninf”, exposed uninfected; “un”/”uninf”, uninfected; “inf”, infected.

Macrophages represented a small percentage of the total cells in this analysis, which is expected from tonsil explant-derived cells. No infected macrophages were present; however, exposed uninfected macrophages were compared to unexposed macrophages and were analyzed for differential gene expression (**Figure 7A**). The following enrichment analysis represented several pathways related to viral infection and immune signaling (**Figure 7B**; **Supplemental Table 6**), including Leishmania infection (R-HSA-5653656). This gene set includes NOD-like receptors (NLRs), cytosolic pattern recognition receptors upstream of inflammasomes that are activated by microbial ligands or danger signals, NLRP3, NLRP1, and NLRC4; ASC (apoptosis-associated speck-like protein containing a CARD), an adaptor protein that links activated NLRs to pro-caspase-1, the protease that cleaves and activates the pro-inflammatory cytokines IL-1β and IL-18; Caspase-1, the protease that is activated by inflammasomes and cleaves pro-IL-1β and pro-IL-18 to produce the mature cytokines; IL-1β and IL-18, pro-inflammatory cytokines that are produced in response to inflammasome activation.

**Figure 7C** shows that exposed uninfected macrophages displayed high levels of the five measured inflammasome genes (*P2RX7, NLRP3, PYCARD, CASP1*, and *IL1B*). No other cell category demonstrated a similar pattern. A dot plot showing the expression of these inflammasome genes in all sub-cell types is shown in **Supplemental Figure 5**, confirming this trend. Similarly, when visualized *via* violin plot, each gene was increased in exposed uninfected macrophages compared to unexposed macrophages (**Figure 7D**). These findings suggest that macrophages play a crucial role in developing HIV-related inflammation, indicating that exposure to HIV leads to significant changes in inflammatory signaling, even without productive infection in that cell type.

Other cell categories were separately analyzed for differential gene expression, including B cells (**Supplemental Figure 6**; **Supplemental Tables 7, 8**), ILCs (**Supplemental Figure 7**; **Supplemental Tables 9, 10**), DCs (**Supplemental Figure 8**; **Supplemental Tables 11, 12**), and epithelial cells (**Supplemental Figure 9**, **Supplemental Table 13**). The expression pattern of various B cell stage markers in infected B cells compared to uninfected B cells, such as the almost absent expression of CD19, also seems to indicate that the very few B cells that were identified as infected may be a different type of B cell than uninfected ones or may even have been misannotated (**Supplemental Figure 6A**). Interestingly, B cells showed a less pronounced difference between the infected and exposed uninfected populations, as shown in **Supplemental Figures 6B, C**, where both differential gene expression analyses resulted in elevated oxidative phosphorylation genes. This may reflect a higher baseline metabolic state of B cells and confirm that productive infection is unlikely, as there is a lack of significant metabolic shift between the intermediate and high viral transcript levels.

ILCs showed enrichment in gene sets primarily associated with protein synthesis and folding (**Supplemental Figure 7**), and DCs exhibited gene sets related to cellular homeostasis (**Supplemental Figure 8**). There were no clear patterns, similarly consistent with a lack of significant difference between cells containing intermediate and high viral transcript levels.

Finally, as shown in **Supplemental Figure 9**, epithelial cellsexpressed various gene sets related to transcription regulation and signaling. Only exposed uninfected and unexposed populations were compared here, as no infected epithelial cells were present.

### Differential gene expression demonstrates profound distinctions between infected T cells and macrophages

Thus far, our observations have distinguished infected T cells as having high levels of oxidative phosphorylation specific to cells containing high levels of HIV-1 transcript, indicative of infection. By contrast, macrophages that are not infected showed high levels of NLRP3 inflammasome genes, suggesting that infected cells can activate related inflammatory signaling.

**Figure 8:**
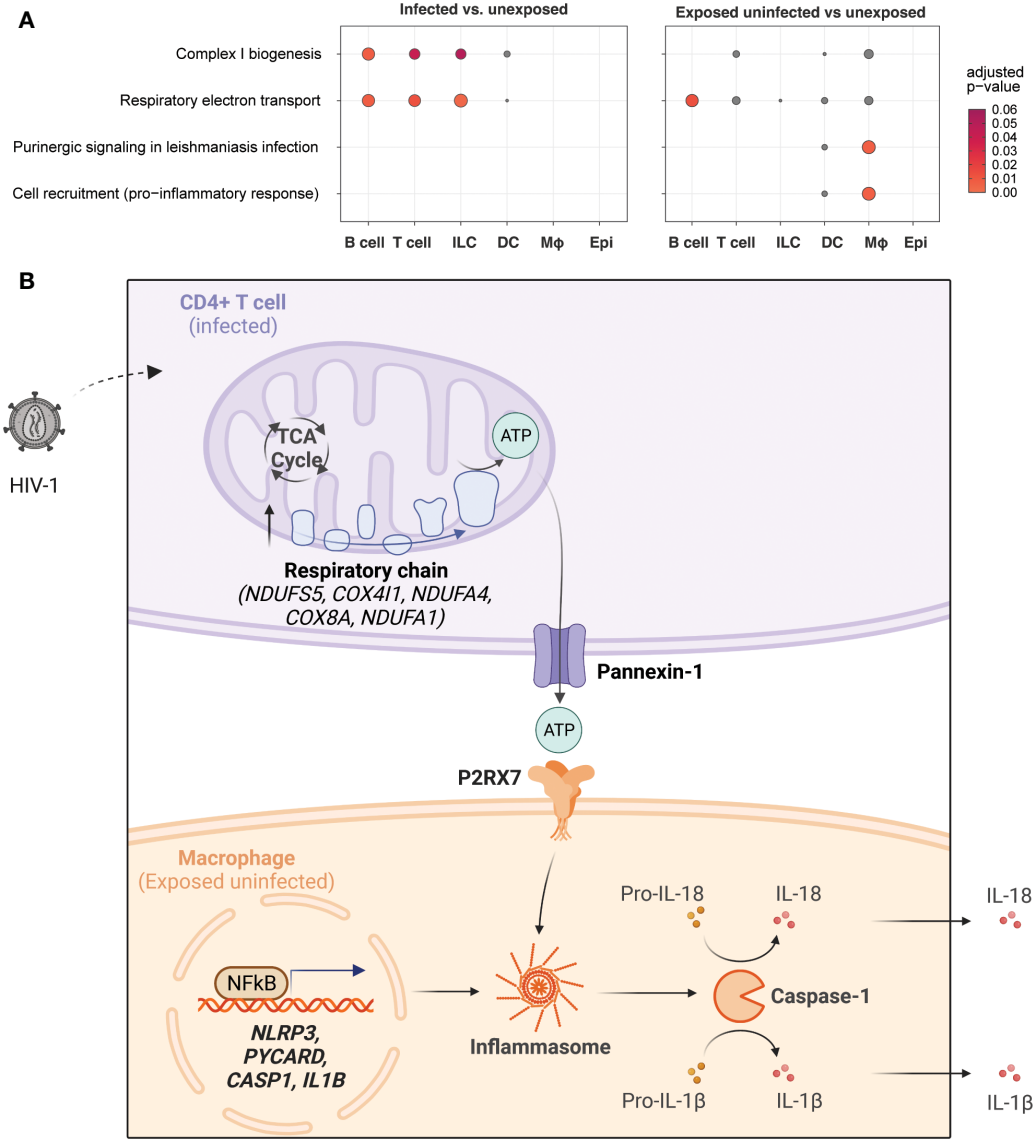
HIV-1 infection promotes oxidative phosphorylation in infected CD4+ T cells and NLRP3 inflammasome activation in macrophages.Dot plots illustrating enrichment of key oxidative phosphorylation and inflammasome signaling gene sets by cell type in infected cells vs. unexposed cells (left) and in exposed uninfected cells vs. unexposed cells (right). Oxidative phosphorylation gene sets include Complex I biogenesis (R-HSA-6799198) and Respiratory electron transport (R-HSA-61105). Inflammasome signaling gene sets includePurinergic signaling in leishmaniasis infection (R-HSA-9660826) and Cell recruitment (pro-inflammatory response) (R-HSA-9664424). The expression of genes related to oxidative phosphorylation is enriched in infected lymphocytes, while genes related to inflammasome signaling are primarily enriched in exposed uninfected macrophages. Dot color and size are proportional to the adjusted enrichment p-value **(A)**. A proposed interaction model between HIV-1 infection, oxidative phosphorylation, and inflammasome pathway signaling in different cell types. HIV-1 infection of CD4^+^ T cells leads to increased glycolysis, tricarboxylic acid cycle (TCA cycle), and oxidative phosphorylation (electron transport chain), resulting in increased adenosine triphosphate (ATP) production. This activates purinergic receptor (P2RX7) signaling in macrophages, leading to activation of the NLRP3 inflammasome and caspase-1 with the release of inflammatory cytokines (e.g., IL-18, IL-1β) **(B)**. Upregulated genes are indicated in bold. Created with BioRender.com.

**Figure 8A** summarizes these findings with a dot plot comparing the enrichment of gene sets of key representative pathways by cell category for infected vs. unexposed cells (left) and exposed uninfected vs. unexposed cells (right). The oxidative phosphorylation pathways such as Complex I biogenesis (R-HSA-6799198) and Respiratory electron transport (R-HSA-611105) were most significant in infected B cells, T cells, and ILCs. While those pathways were also elevated in B cells in the exposed uninfected group, this distinguished infected T cells as uniquely impacted by HIV-1 productive infection. By contrast, the inflammasome signaling pathways, such as Purinergic signaling in leishmaniasis infection (R-HSA-9660826) and Cell recruitment (pro-inflammatory response) (R-HSA-9664424), were most significant in the exposed uninfected macrophages. **Supplemental Figure 10** expands the included cell types of this dot plot and similarly shows that oxidative phosphorylation pathways were generally increased in infected B cells and CD4 T cells. In contrast, the inflammasome signaling pathways were enriched in macrophages in the exposed uninfected group. A complete list of DEGs for infected vs. unexposed and exposed uninfected vs. unexposed total cells and by cell categories can be found in **Supplemental Tables 2–11**.

## Discussion

Here we demonstrate how single-cell RNA sequencing analysis of a human tonsil explant model can characterize key immune cell subsets involved in HIV-1 infection and induced transcriptomic changes not accessible *via* bulk RNA analysis. The most identified immune cell types in the tonsil explants were lymphocytes, including B cells, CD4^+^ T cells, and CD8^+^ T cells. HIV-1 exposure resulted in approximately 2.5% infection as measured by flow cytometry mCherry expression, consistent with reported rates in similar *ex vivo* tonsil explant models (57, 58). Infected cells were found most prominently in the CD4^+^ T cell compartment, consistent with the understanding that CD4^+^ T cells are most permissive to HIV-1 infection. Among the infected cells, a small population included B cells which are not expected to be productively infected. There are several possible explanations for this finding. The first is that it is possible that those cells are not, in fact, B cells. They represent a minor population of the total B cell population identified by cluster analysis based on the expression of CD19, CD27, CD24, CD38, MME, CR2, and MS4A1. These seven genes were the most distinctive in characterizing the common features of those cells identified as naive B cells, memory B cells, GC B cells, and plasma cells. It is important to note that these markers are not exclusive to B cells and that HIV-1 infection of CD4 T cells might shift expression such that these infected cells might be T cells. The following markers have been described to various extents in CD4 T cells that might impact the characterization: CD19, CD27, CD24, CD38, CR2, and MS41 (59–65). It is important to note that much of the literature on these markers is based on protein expression and not transcriptomics. Together with how transcriptional changes are impacted by HIV-infected, there may be confounding variables (66). Other potential explanations for these markers include the presence of cells that express alternative HIV receptors or engage in antibody-dependent cellular phagocytosis while harboring HIV RNA. It is worth noting that the macrophage and epithelial cell compartments did not contain any infected cells.

The upregulation of both oxidative phosphorylation genes and pannexin-1 in infected T cells suggests an important role for these processes in the immune response to viral infections. The high levels of oxidative phosphorylation in infected T cells and the upregulation of pannexin-1 may allow these cells to communicate with nearby cells and mediate inflammatory signaling. The fact that pannexin-1 is more ubiquitously expressed than pannexin-2, which is more prominently expressed in the central nervous system (67), may explain why the effect of transcriptional upregulation in infected cells is more pronounced in pannexin-1.

A possible model to integrate and interpret our findings is shown in **Figure 8**. A productively HIV-1-infected CD4^+^ T cell may undergo transcriptional upregulation of metabolism, notably the TCA cycle and oxidative phosphorylation. This results in increased ATP production, which travels out through pannexin-1 in the infected CD4^+^ T cell and is recognized by the P2RX7 receptor on the surface of nearby macrophages. In concert with TLR signaling to activate pro-IL-1B and pro-IL-18 transcription, ATP-mediated purinergic signaling can trigger the NLRP3 inflammasome to stimulate caspase-1 activation and secretion of IL-1β and IL-18. This may result in pyroptotic cell death or cytokine secretion that can produce a systemic immune response and potentiate systemic inflammatory activation. Further work to investigate this and other potential models will be necessary to explain the increased oxidative phosphorylation in infected T cells.

Limitations to this study include tonsil model infection rates that are likely higher than what occurs *in vivo*. The virus used in these experiments is an X4-tropic virus, whereas transmitting founder viruses are likely to be R5-tropic and do not replicate well in this tonsil explant model. There was inter-donor variability in infection levels, representing individual-specific baseline levels of immune activation. The study focused on viable cells, which are disproportionately lymphocytes. The human tonsil explant model must better represent a critical observed cell type in the interplay between infected CD4+ T cells and other immune cells in the macrophages. None of the macrophages had high levels of infection; all analysis for macrophages was performed comparing uninfected to exposed. The nature of the high levels of HIV-1 transcript is unclear, as described above. The *ex vivo* nature of our model may demonstrate phenotypes that differ from those that arise from natural infection

, such as the enrichment of lymphocytes in this model and lower representation of other cell types, such as myeloid cells, epithelial cells, and other stromal cells. Another possible limitation of tonsil explant models is variability in survival between cell types, thereby leading to over- and under-representation of immune cell subsets. Further variability arises from the use of human tissue donors. Heterogeneity can be seen in infection levels and transcriptomic profiles between individuals. Our tonsil model also uses *ex vivo* infection to model *in vivo* pathogenesis. Viral loads in these infected tonsil explants are likely significantly higher than in patients.

B cells are not considered to be permissive to HIV-1 infection; B cell infection by HIV-1 has been reported but with lower efficiency than in T cells (68–70). Similarly, ILCs are not thought capable of HIV-1 productive infection; however, some reports demonstrate that NK cells can be infected (71–73), particularly with X4-tropic virus, whose clone was used in this study. It is also possible that B cells and ILCs harbor HIV-1 transcripts due to endocytosis or have virus particles bound to their cell surface. In both cases, HIV transcripts may be detected in ILCs even if they are not producing new viruses.

Single-cell RNA-sequencing identified 71% of exposed cells to exhibit low and widely heterogeneous levels of HIV-1 transcript. The biological significance of these transcripts, if any, and the discordance between viral transcript and protein expression remains unclear. One potential explanation for the low-level HIV-1 transcript expression in exposed uninfected cells is as an artifact, such as detecting virions adherent to cells’ surfaces despite single-cell processing. As the low transcript levels are specific to the exposed uninfected cells, this may reflect a “bystander” status – close physical proximity to an infected cell producing large amounts of HIV-1 transcript. A second possibility is that the exposed uninfected cells harboring low to intermediate levels of HIV-1 transcript may represent non-productive or latent infection due to impaired integration, transcription, and translation. Multiple groups have reported profound variations in HIV-1 transcription and protein expression in cell lines infected with HIV-1 and primary cells from HIV-1-infected donors (74–78). Leon-Rivera reported a 1:1 correlation between HIV Gag and HIV transcript expression amongst monocytes exposed to HIV-1 (79). Heterogeneity in HIV-1 transcription is related to cell-specific modifications in late and post-transcription (e.g., transcript elongation, polyadenylation, splicing) (75), differential transcription of proviruses due to features of the integration sites (e.g., epigenetic, architectural) (76), and cell-specific responsiveness to latency and reactivation (77). Recently, Lim et al. reported that an activated monocyte cell line infected with a DHIV3-mCherry pseudovirus demonstrated heterogeneous HIV-1 transcript expression levels and discordance between viral transcript and protein expression. 8% of these monocytes expressed high levels of HIV-1 transcript, viral proteins, and HIV-associated marker proteins. 50% of the cells expressed low transcript levels but no viral or marker proteins (78). The latter cells were postulated to represent those containing HIV-1 mRNA transcribed from pre-integration cDNA complexes (i.e., non-integrated viral genomes) (78). Expanding upon a tonsil explant model by incorporating primers specific to pre-integration cDNA will help elucidate the biological significance of cell-specific immune responses, MALT cytoarchitecture, and the nature of the exposed uninfected cells described here.

In probing the transcriptomic modifications induced by HIV-1 exposure and infection in a human tonsil model, we demonstrate that different cell types exhibit variable patterns of transcriptional regulation in response to HIV-1 exposure. In contrast to our observations, Lim et al. observed no transcriptomic differences in cells with or without detectable HIV-1 transcript (78). Using the human tonsil model, we observed key differences in gene expression among single cells with varying HIV-1 transcript expression levels. Targeted assessment of NLRP3 inflammasome pathway genes demonstrated the most significant expression of *P2RX7*, *NLRP3*, *CASP1*, *PYCARD*, and *IL1B* in the macrophage compartment, notably in those exposed uninfected cells compared to unexposed cells.

Broader differential expression stratified by HIV-1 transcript expression levels confirmed the enrichment of inflammatory pathway genes in macrophages and, notably, identified additional enrichment of respiratory chain pathway genes in CD4^+^ T cells. The role of the NLRP3 inflammasome in HIV-1 pathogenesis has been described in multiple cell types, including macrophages and lymphocytes, as a mediator of pro-inflammatory signaling and pyroptotic programmed cell death (14–34, 36, 37, 67, 80–109). The role of oxidative phosphorylation has been described in clinical settings as related to mitochondrial dysfunction (110–113). HIV-1 can increase metabolism and immune cell oxidative stress in PWH (114). Glucose transporter 1 (GLUT1) is upregulated in monocytes and CD4+ T cells of PWH (115–118). Recently, Guo et al. (119) analyzed CD4^+^ T cells from PWH and observed that elevated oxidative phosphorylation (OXPHOS) pathways are associated with poorer disease outcomes. They further demonstrated that metformin treatment, a drug targeting the mitochondrial respiratory chain gene complex I, reduced HIV-1 replication in human CD4^+^ T cells and humanized mice. Notably, the upregulation of Pannexin1 in infected T cells suggests that ATP generated through elevated oxidative phosphorylation can be released through Pannexin1 and detected by nearby cells. These observations imply that HIV-1 may differentially induce oxidative phosphorylation and NLRP3 inflammasome signaling pathways in a cell subtype-specific manner.

Future studies should probe the spatial interactions of macrophages with T cells to understand their geographical association in the lymphoid environment. Our laboratory has observed that HIV-induced inflammatory cytokine stimulation does not occur in PBMCs or tonsil-derived single-cell culture (120), suggesting that native lymphoid structure is required to mediate these inflammatory signaling pathways. This may relate to the close apposition of cells, epithelial and stroma cell contributions, and the 3D architecture of the tissue.

Our study highlights the differential roles of HIV-infected CD4+ T cells and macrophages in mediating inflammatory signaling in a human tonsil explant model. Our results demonstrate a cell type-specific transcriptomic response induced by HIV-1 exposure and productive infection. We hypothesize that activation of oxidative phosphorylation in cells expressing high levels of HIV-1 transcript (CD4+ T cells) induces high levels of ATP efflux that is subsequently detected by macrophage purinergic receptors (**Figure 8B**). This may trigger a macrophage compartment signaling cascade leading to NLRP3 inflammasome activation and inflammatory cytokine production, as shown in **Figure 8**. Our findings suggest that targeting the specific cell types and pathways involved in HIV-mediated inflammation could be a potential strategy to develop more effective therapeutic interventions to alleviate chronic inflammation in HIV-infected individuals.

Further studies are needed to understand the complex interplay between immune cell types and signaling pathways in HIV-mediated inflammation and to develop more targeted and personalized treatment strategies for HIV infection. Future directions of this study include investigating the impact of HIV-1 on other cell types within the tonsil tissue, such as dendritic cells, follicular helper T cells, and regulatory T cells, to gain a complete picture of the immune response to infection. The potential for therapeutic interventions to mitigate the inflammatory response induced by HIV-1 exposure and productive infection, such as targeting ATP efflux or the NLRP3 inflammasome, will be examined. The implications of the observed cell type-specific transcriptomic response in other HIV-1 infection models, including those in different tissue types and animal models, will also be explored. Furthermore, the potential for crosstalk between infected and uninfected cells in the context of HIV-1 infection, and the impact of such interactions on the inflammatory response, will be investigated.

## Materials and methods

### Study participants, sampling protocols, and experimental approach

Human tonsil explants were obtained from healthy tonsillectomy patients and dissected into tissue blocks cultivated on collagen rafts, as previously described (50, 120). Briefly, palatine tonsil tissue was dissected into nine 1 mm explants which were subsequently cultured and exposed to HIV-1_NL-CI_, a CXCR4-tropic fluorescent reporter virus with mCherry in place of *nef* where *nef* is expressed on an IRES, as previously described (56). Media was removed on days 2, 5, and 8 following HIV-1 NL-CI exposure. Cells were collected and washed, as shown in **Figure 1A**, which describes the flow of sample collection through single cell isolation and cDNA generation, single-cell sequencing, data pre-processing, and analysis, including cell identification and cluster annotation, compositional analysis, network analysis, and differential expression. Viability and infection were quantified by flow cytometry with LIVE/DEAD stain and mCherry expression, respectively (**Figures 1B, C**). Infection was observed over eight days with stable viability and demonstrated productive infection of 2.45% of exposed cells by day 8.

### Cell lines

293T cells (American Type Culture Collection, Co#: CRL-3216) were propagated at 37°C and 5% CO_2_ in Dulbecco’s Modified Eagle Medium (DMEM; Sigma) containing 10% fetal bovine serum (FBS; Gibco), 100 U/mL penicillin, 10U/mL streptomycin, and two mM glutamine (Gibco).

### Virus production

HIV-1 NL-C1 is a fluorescent reporter clone of HIV_NL4-3_ that expresses mCherry in place of *nef*, expressed *via* an internal ribosome entry site (IRES). Cell-free HIV-1_NL-CI_ was produced by lipofection of 293T cells (PolyJet, Signagen). Supernatants were harvested 48 hours after transfection and clarified by centrifugation at 100,000 x *g* at four °C for two h (Sorvall ST 40R centrifuge; Thermo Fisher Scientific) followed by 0.45-μm filtration. HIV-1 p24 antigen concentration in the supernatant was quantified using enzyme-linked immunosorbent assay (ELISA) with coating antibody D7320 (sheep anti-HIV-1-p24 gag; Aalto Bio Reagents) as previously described (120). Single-use aliquots were stored at −80°C.

### Preparation, *ex vivo* infection, and human tonsil explant tissue block processing

Human tonsils were collected within several hours of routine tonsillectomies performed by B. Tweel at the Mount Sinai Health System in New York City under an Institutional Review Board-approved protocol #20-00930. Human tonsil explants were dissected into 1-mm tissue blocks that were plated on top of a collagen sponge (Surgifoam; Ethicon) and maintained in RPMI 1640 medium (Life Technologies) containing 15% fetal bovine serum (FBS; Gibco), 2mM GlutaMax (Life Technologies), 1 mM sodium pyruvate (Corning), 1% minimal essential medium (MEM) nonessential amino acids (Corning), 2.5 μg/ml amphotericin B (HyClone), 50 mg/ml gentamicin sulfate (Corning), and 0.3 mg/ml Timentin (bioWORLD) as previously described (50, 120). The human tonsil explant tissue blocks were left uninfected or individually inoculated with 5 μL of HIV-1_NL-CI_ (equivalent to 3.24 ng of p24). Complete media changes were performed post-infection on days 2, 5, and 8. Suspension cells for flow cytometry and scRNA -seq were harvested by centrifugation (2000 x g, 5 min) of tonsil explant supernatants at indicated time points.

### Flow cytometry and gating strategy

Collected suspension cells from tonsil explants were washed twice with phosphate-buffered saline (PBS) in 96-well round-bottom plates. Cells were stained with a fluorescent viability dye (LIVE/DEAD Fixable Violet stain; Thermo Fisher Scientific) at a concentration of 1:1000 in PBS for 20 min at 4C. Stained cells were washed with PBS supplemented with two mM EDTA and 0.5% bovine serum albumin and subsequently fixed with 2% paraformaldehyde. Flow cytometry was conducted with an Attune NxT Flow Cytometer (Thermo Fisher Scientific). Cells were initially identified by forward and side scatter areas (FSC-A/SSC-A). Inclusion gates for viability and infection as measured by HIV-1_NL-CI_ mCherry expression were determined by unstained and uninfected control populations, respectively. Data were exported and analyzed with FlowJo software (BD).

### Single-cell RNA sequencing

Tonsil samples from seven donors were processed for single-cell RNA sequencing (scRNA-seq) on day eight following exposure to HIV-1_NL-CI_. Suspension cells in tonsil explant supernatants were harvested *via* centrifugation of supernatants at 2000 x g for 5 min. The viability of collected cells was assessed using flow cytometry as described above, and debris-free suspensions of >80% viability were deemed suitable for scRNA-seq. scRNA-seq was performed on these samples using the Chromium platform (10x Genomics, Pleasanton, CA) with the 3’ gene expression (3’ GEX) V3 kit, using an input of ~10,000 cells. Briefly, Gel-Bead in Emulsions (GEMs) were generated on the sample chip in the Chromium controller. Post-GEM reverse transcription (RT) cleanup was performed, and barcoded cDNA extracted from the GEMs was amplified for 12 cycles. Amplified cDNA was fragmented and subjected to end-repair, polyadenylation, adapter ligation, and 10X-specific sample indexing following the manufacturer’s protocol. Libraries were quantified using Bioanalyzer (Agilent) and QuBit (Thermo Fisher Scientific) analysis. Libraries were sequenced in paired-end mode on a NovaSeq instrument (Illumina), targeting a depth of 50,000-100,000 reads per cell. Sequencing data were aligned and quantified using the Cell Ranger Single-Cell Software Suite (version 3.0, 10x Genomics) against the provided custom GRCh38/HIV-1_NL-CI_ human reference genome. HIV-1 transcripts were identified as sequenced 10X single-cell RNASeq data and were de-multiplexed with bcl2fastq v2.20.0 (invoked by the mkfastq command in various Cell Ranger versions). De-multiplexed data were processed by Cell Ranger (v6.0.1 and v6.1.0). Reads were aligned to a joint reference that consists of 10X’s GRCh38-2020-A Human reference and a custom HIV reference whose regions with known features are marked as exonic. The pipeline grouped and de-duplicated reads confidently mapped to the transcriptome by 10X cellular barcodes and UMIs (Unique Molecular Identifiers), then summarized counts into feature-barcode matrices. Downstream analyses, such as graph-based clustering and differential expression analysis/visualization, were performed using the Loupe Cell Browser (10x Genomics) and Seurat (121–123), as illustrated in **Figure 1A**.

*scRNA-seq analysis.* The R-based package Seurat (version 4.0.5) (124) was used to process the scRNA-seq data. Genes detected in less than 0.5% of cells were excluded from the analysis. Cells with an expression of < 1000 or > 40000 total molecules, < 100 or > 6000 unique genes, and > 15% mitochondrial genes were removed for quality control before analysis. The normalized datasets of each treatment group (HIV unexposed and HIV exposed) were integrated through the Seurat RPCA integration method, using unexposed samples as references. The final data set for further analysis comprised 17564 genes and 47686 cells derived from seven tonsil donors. Normalized transcripts were scaled and centered using built-in Seurat functions. For dimensionality reduction and clustering, variable transcripts were calculated based on standardized feature values using a local polynomial regression model’s observed mean and expected variance. On the resulting variable transcripts, 30 principal components were computed and used as input for uniform manifold approximation and projection (UMAP) dimensionality reduction. A shared nearest neighbor (SNN) graph was constructed for clustering analysis, and the modularity function was optimized using the Leiden algorithm. Cell type category annotation was performed using cell type-specific marker expression, as follows: B cells (*CD19, CD79A, MS4A1*), T cells (*CD3D, CD8A, CD4*), ILCs (*KLRC1, GNLY*), DCs (*CCR7*), macrophages (*CD14, CD68, ITGAX*), and epithelial cells (*CDH1, CLDN4, KRT8*). B and T cell compartments were further stratified using more specific markers (B cells (*CD19, CD27, CD24, CD38, MS4A1, CR2*), T cells (*CD3D, CD8A, CD8B, CD4, CCR7, SELL, LRRN3, IL7R, IL2RA, CTLA4, ICOS, EOMES, KLRG1, GZMB, GZMK, NKG7, GNLY, PRF1, FOXP3, CD38, ITGAE, TNFRSF4, BCL6, TCF7, CD28, LEF1*) as well as an additional external scRNA-seq data set of human tonsils (125).

The 2.45% of HIV-exposed cells with the highest HIV transcript levels were presumed to be productively infected, as determined by FACS analysis (**Figure 1**). Overall HIV transcript distribution in mCherry-sorted cells (**Figure 3C**) verified this estimate, and these cells were classified as “Infected.” The remaining HIV-exposed cells were identified as “Exposed uninfected,” and cells stemming from samples with no prior virus contact as “Unexposed.” Differential expression analysis was performed using the Seurat function *FindMarkers()*, specifying the tonsil donor as a latent variable to correct for inter-donor differences and reduce the impact of confounding variables or batch effects. Gene set enrichment of the resulting differentially expressed genes in the Reactome pathways (126) was performed using the ReactomePA R package (127), which aggregates the per-gene statistics across genes within a gene set, therefore making it possible to detect situations where all genes in a predefined set change in a small but coordinated way.

## Data availability statement

The data presented in the study are deposited in the Gene Expression Omnibus (GEO) repository, accession number GSE232477. https://www.ncbi.nlm.nih.gov/geo/query/acc.cgi?acc=GSE232477.

## Ethics statement

The studies involving human participants were reviewed and approved by The Program for the Protection of Human Subjects Institutional Review Board at the Icahn School of Medicine atMount Sinai under STUDY-20-00930. The patients/participants provided their written informed consent to participate in this study.

## Author contributions

TFr, TFo, and SS performed experiments. BT collected the patient samples. TFr, CZ, NS, KB, and TS analyzed the results and wrote the paper. KB and TS conceived the approach. TFr, CZ, and NS share the first author position because each contributed substantially to the experimentation, data acquisition, and analysis. KB and TS share the last author position because they co-conceived the approach and share supervision of the teams conceiving, implementing, experimenting, data acquisition, analyzing, and writing this manuscript.

## Supplementary material

Supplementary Figure 1 Distinct cell types in human tonsil explants. Dot plot showing cell type identification and cluster annotation were based on known immune and non-immune cell marker gene expression patterns. Dot size is proportional to the percentage of cells within a compartment. The dot color indicates the average expression across the cluster (red = high, blue = low).

Supplementary Figure 2 Characterization of HIV-1 infection by donor. UMAP plots of the populations are color-coded by exposure and infection **(A)**, and bar plots of each donor are indicated by exposure and infection status **(B)**. Ridge plots are shown for HIV infection by tonsil donors **(C)**.

Supplementary Figure 3 Impact of HIV-1_NL-CI_ exposure on cell type. Stacked bar plots illustrate relative frequencies of cell types in the merged, integrated dataset, stratified by tonsil donor and HIV-1 exposure **(A)**. Ridge plots show the distribution of HIV-1 transcript expression in each cell type by HIV-1 exposure category: unexposed, exposed uninfected, and infected **(B)**. Bar plots show the frequency of HIV-1 infection by cell type. Error bars denote the standard error of the mean **(C)**.

Supplementary Figure 4 Patterns of T cell expression with HIV-1 infection and exposure. Volcano plot of genes differentially expressed **(A)**, and bar plot illustrating the top 15 Reactome database gene sets enriched in exposed uninfected T cells vs. exposed T cells **(B)**. Colors denote significance and fold change cutoffs: red = absolute log2(fold change) ≥ 0.6 and adjusted p-value < 0.05, pink = absolute log2(fold change) < 0.6 and adjusted p-value <0.05, orange = absolute log2(fold change) ≥ 0.6 and adjusted p-value >0.05, grey = absolute log2(fold change) < 0.6 and adjusted p-value >0.05. Gene sets with adjusted p-value <0.05 were considered significant **(A)**. A positive Normalized Enrichment Score (NES) value indicates enrichment **(B)**. Dot plots demonstrate the expression of 5 oxidative phosphorylation genes in each sub-cell type, comparing unexposed, exposed uninfected, and infected cells. Dot size is proportional to the percentage of cells within a group. The dot color indicates the average expression across the group (red = high, blue = low) **(C)**.

Supplementary Figure 5 Inflammasome gene expression by cell type. Dot plots demonstrate the expression of five NLRP3 inflammasome genes by cell type for unexposed cells, exposed uninfected, and infected. Dot size is proportional to the percentage of cells within a group. The dot color indicates the average expression across the group (red = high, blue = low).

Supplementary Figure 6 Differential expression by HIV-1_NL-CI_ infection and exposure in B cells. Violin plots of various B cell marker gene expression are shown in B cells **(A)**. Volcano plots of genes differentially expressed in infected cells vs. unexposed cells **(B)** and exposed uninfected cells vs. unexposed cells **(D)**. Colors denote significance and fold change cutoffs: red = absolute log2(fold change) ≥ 0.6 and adjusted p-value < 0.05, pink = absolute log2(fold change) < 0.6 and adjusted p-value <0.05, orange = absolute log2(fold change) ≥ 0.6 and adjusted p-value >0.05, grey = absolute log2(fold change) < 0.6 and adjusted p-value >0.05 **(B, D)**. Bar plots illustrate the top 15 Reactome database gene sets enriched in infected cells vs. unexposed cells **(C)** and exposed uninfected cells vs. unexposed cells **(E)**. Gene sets with adjusted p-value <0.05 were considered significant. A positive Normalized Enrichment Score (NES) value indicates enrichment **(C, E)**.

Supplementary Figure 7 Differential expression by HIV-1_NL-CI_ infection and exposure in innate lymphoid cells (ILC). Volcano plots of genes differentially expressed in infected cells vs. unexposed cells **(A)** and in exposed uninfected cells vs. unexposed cells **(C)**. Colors denote significance and fold change cutoffs: red = absolute log2(fold change) ≥ 0.6 and adjusted p-value < 0.05, pink = absolute log2(fold change) < 0.6 and adjusted p-value <0.05, orange = absolute log2(fold change) ≥ 0.6 and adjusted p-value >0.05, grey = absolute log2(fold change) < 0.6 and adjusted p-value >0.05 **(A, C)**. Bar plots illustrate the top 15 Reactome database gene sets enriched in infected cells vs. unexposed cells **(B)** and exposed uninfected cells vs. unexposed cells **(D)**. Gene sets with adjusted p-value <0.05 were considered significant. A positive Normalized Enrichment Score (NES) value indicates enrichment **(B, D)**.

Supplementary Figure 8 Differential expression by HIV-1_NL-CI_ infection and exposure in dendritic cells. Volcano plots of genes differentially expressed in infected cells vs. unexposed cells **(A)** and in exposed uninfected cells vs. unexposed cells **(C)**. Colors denote significance and fold change cutoffs: red = absolute log2(fold change) ≥ 0.6 and adjusted p-value < 0.05, pink = absolute log2(fold change) < 0.6 and adjusted p-value <0.05, orange = absolute log2(fold change) ≥ 0.6 and adjusted p-value >0.05, grey = absolute log2(fold change) < 0.6 and adjusted p-value >0.05 **(A, C)**. Bar plots illustrate the top 15 Reactome database gene sets enriched in infected cells vs. unexposed cells **(B)** and exposed uninfected cells vs. unexposed cells **(D)**. Gene sets with adjusted p-value <0.05 were considered significant. A positive Normalized Enrichment Score (NES) value indicates enrichment **(B, D)**.

Supplementary Figure 9 Differential expression by HIV-1_NL-CI_ infection and exposure in epithelial cells. Volcano plots of genes differentially expressed in exposed uninfected cells vs. unexposed cells. Colors denote significance and fold change cutoffs: red = absolute log2(fold change) ≥ 0.6 and adjusted p-value < 0.05, pink = absolute log2(fold change) < 0.6 and adjusted p-value <0.05, orange = absolute log2(fold change) ≥ 0.6 and adjusted p-value >0.05, grey = absolute log2(fold change) < 0.6 and adjusted p-value >0.05. Bar plots illustrate the top 15 Reactome database gene sets enriched in infected cells vs. unexposed cells **(C)** and exposed uninfected cells vs. unexposed cells **(D)**. Gene sets with adjusted p-value <0.05 were considered significant. A positive Normalized Enrichment Score (NES) value indicates enrichment.

Supplementary Figure 10 Inflammasome gene expression by cell type. Dot plots illustrating enrichment of key oxidative phosphorylation and inflammasome signaling gene sets by cell type in infected cells vs. unexposed cells (left) and in exposed uninfected cells vs. unexposed cells (right). Dot color and size are proportional to the adjusted enrichment p-value.
