# Supplementary material for: HIV-1 activates oxidative phosphorylation in infected CD4 T cells in a human tonsil explant model
Source: Front Immunol. 2023 May 30;14:1172938. doi: 10.3389/fimmu.2023.1172938 (PMC10266353; doi:10.3389/fimmu.2023.1172938)
Supplement: Supplementary file 1 [file Table_1.docx]

Supplemental Table 1. The abundance of cell categories and types among infected cells.

| **Cell category** | **% cells of infected cells** | **Cell type** | **% cells of infected cells** |
| --- | --- | --- | --- |
| **B cell** | 5.9 | Naïve B cell | 1.0 |
|  |  | Memory B cell | 4.2 |
|  |  | Plasma cell | 0.7 |
| **T cell** | 90.7 | CD3+ CD19+ lymphocytes | 4.2 |
|  |  | Naïve T cell | 2.7 |
|  |  | CD4 naive/memory T cell | 10.8 |
|  |  | CD4 T cell | 3.9 |
|  |  | CD4 CTL | 16.3 |
|  |  | Tfh | 14.1 |
|  |  | Treg | 18.9 |
|  |  | CD8 naive/memory T cell | 12.4 |
|  |  | CD8 CTL | 0.8 |
|  |  | Cycling T cell | 6.5 |
| **ILC** | 2.0 | ILC | 2.0 |
| **DC** | 1.4 | DC | 1.4 |
| **Macrophage** | 0 | Macrophage | 0 |
| **Epithelial** | 0 | Squamous epithelial cell | 0 |
|  |  | Club epithelial cell |  |
|  |  | Secretory cell | 0 |
