# Supplementary figures and images for: HIV-1 activates oxidative phosphorylation in infected CD4 T cells in a human tonsil explant model
Source: Front Immunol. 2023 May 30;14:1172938. doi: 10.3389/fimmu.2023.1172938 (PMC10266353; doi:10.3389/fimmu.2023.1172938)

Identity

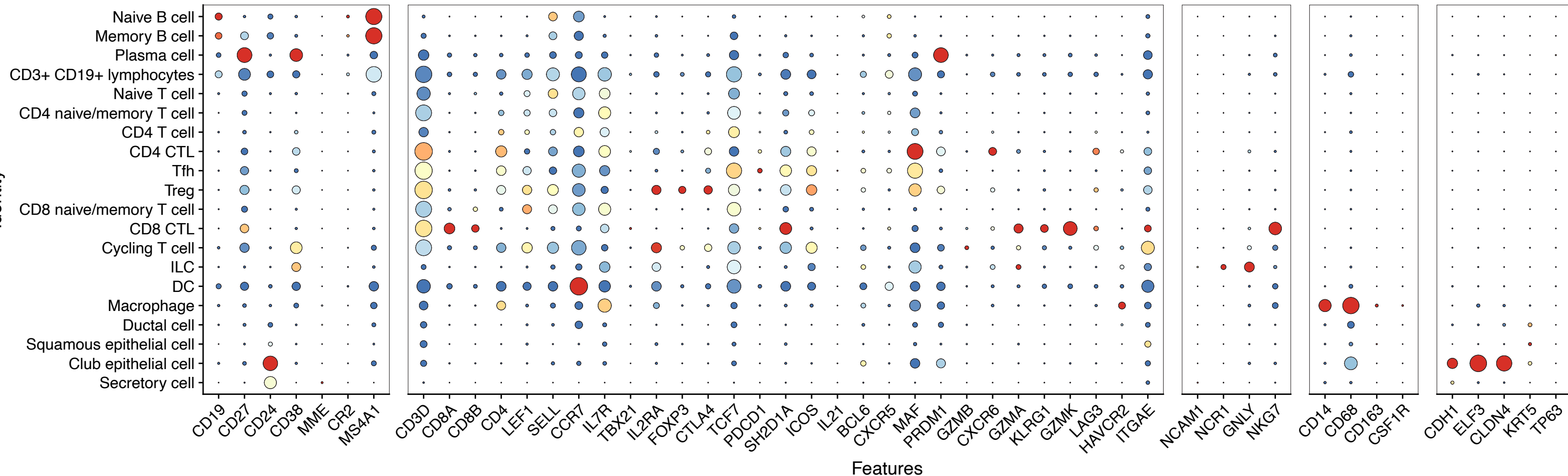

Supplement: Supplementary Figure 1 — Distinct cell types in human tonsil explants. Dot plot showing cell type identification and cluster annotation were based on known immune and non-immune cell marker gene expression patterns. Dot size is proportional to the percentage of cells within a compartment. The dot color indicates the average expression across the cluster (red = high, blue = low). [file Image_1.pdf]

A

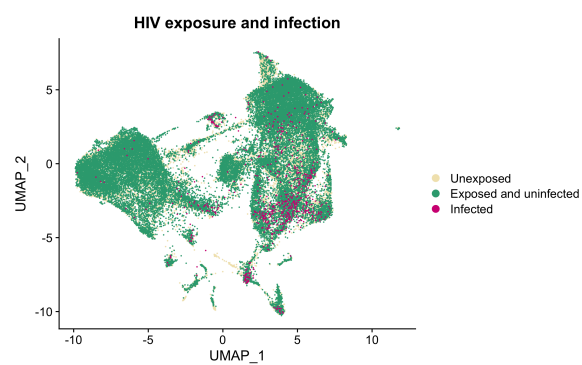

B

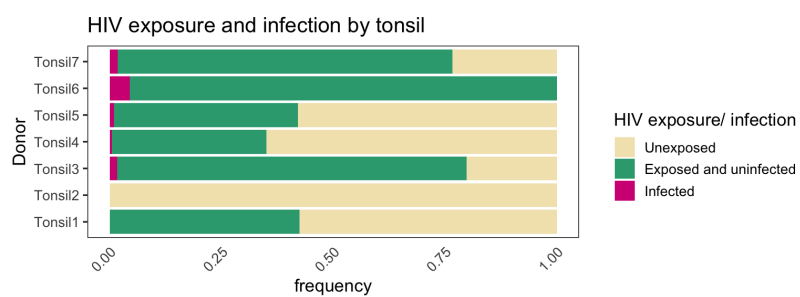

C

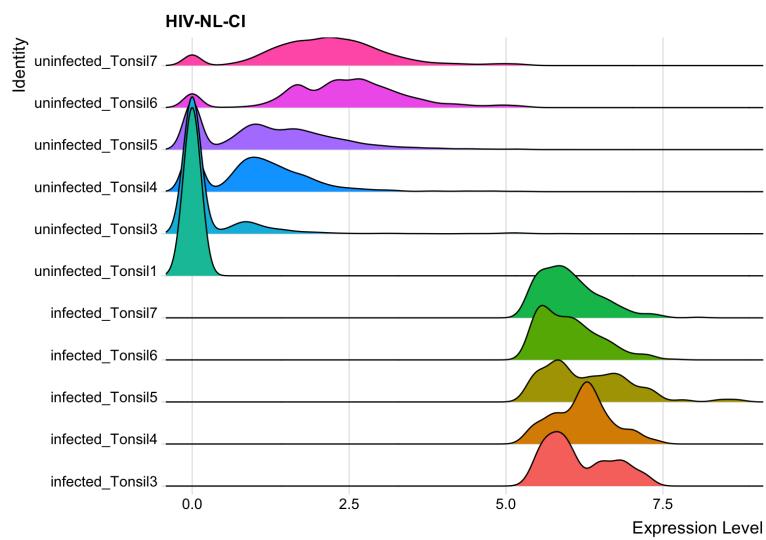

Supplement: Supplementary Figure 2 — Characterization of HIV-1 infection by donor. UMAP plots of the populations are color-coded by exposure and infection (A), and bar plots of each donor are indicated by exposure and infection status (B). Ridge plots are shown for HIV infection by tonsil donors (C). [file Image_2.pdf]

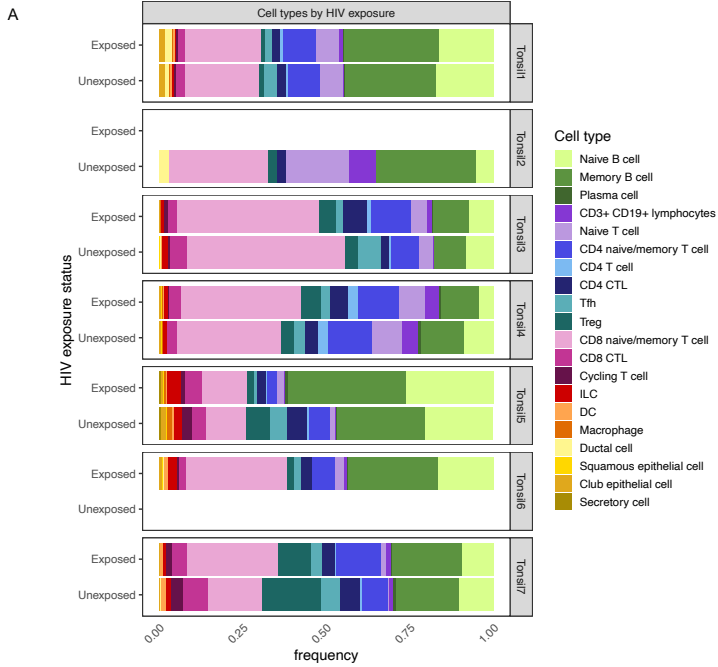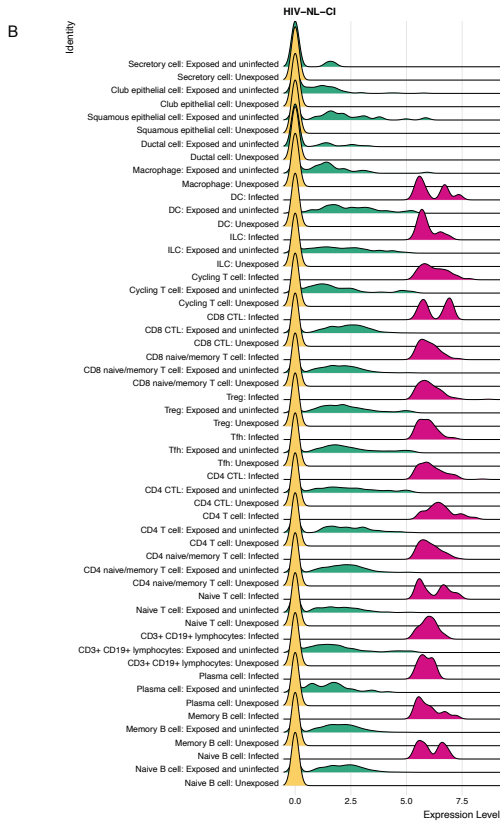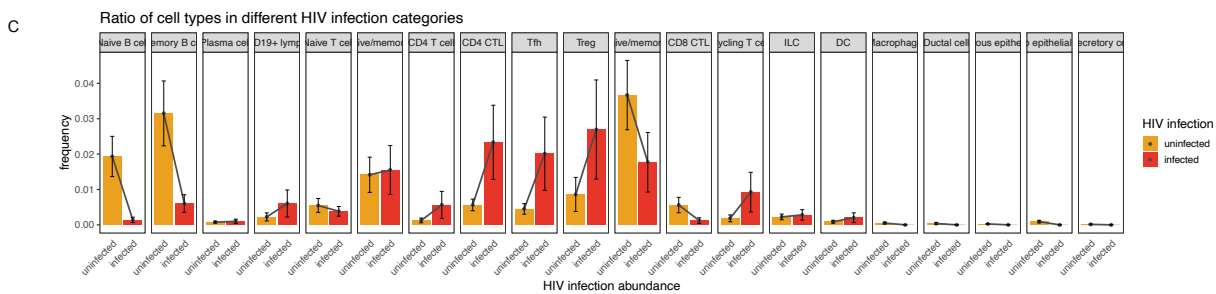

Supplement: Supplementary Figure 3 — Impact of HIV-1NL-CI exposure on cell type. Stacked bar plots illustrate relative frequencies of cell types in the merged, integrated dataset, stratified by tonsil donor and HIV-1 exposure (A). Ridge plots show the distribution of HIV-1 transcript expression in each cell type by HIV-1 exposure category: unexposed, exposed uninfected, and infected (B). Bar plots show the frequency of HIV-1 infection by cell type. Error bars denote the standard error of the mean (C). [file Image_3.pdf]

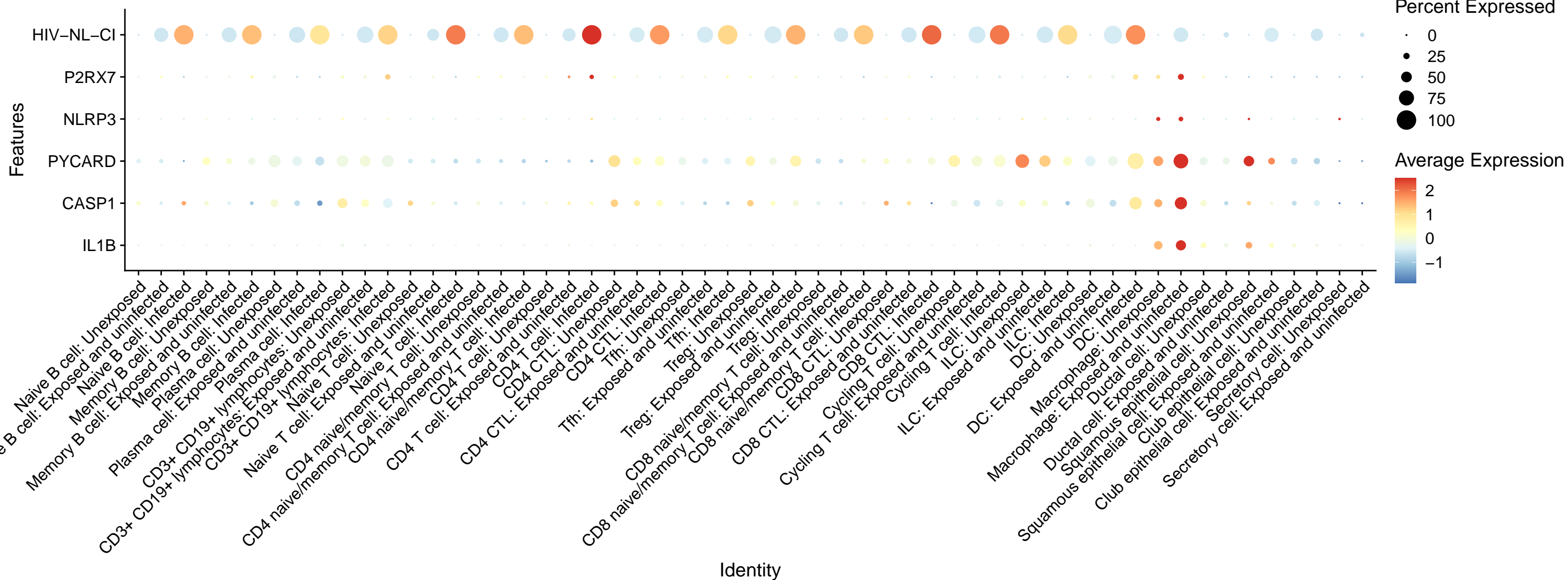

Supplement: Supplementary Figure 5 — Inflammasome gene expression by cell type. Dot plots demonstrate the expression of five NLRP3 inflammasome genes by cell type for unexposed cells, exposed uninfected, and infected. Dot size is proportional to the percentage of cells within a group. The dot color indicates the average expression across the group (red = high, blue = low). [file Image_5.pdf]

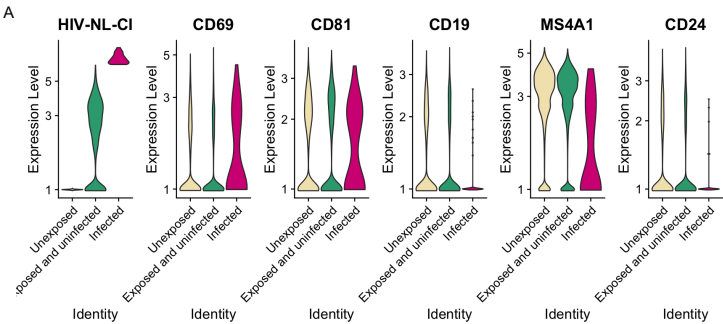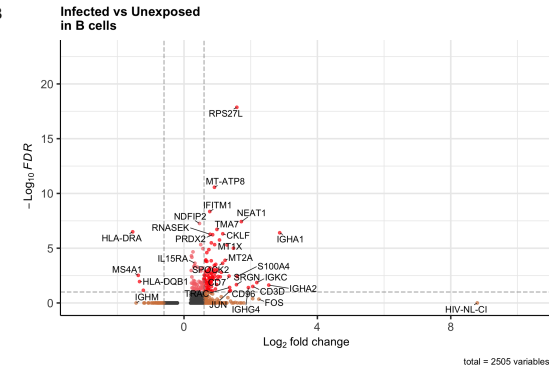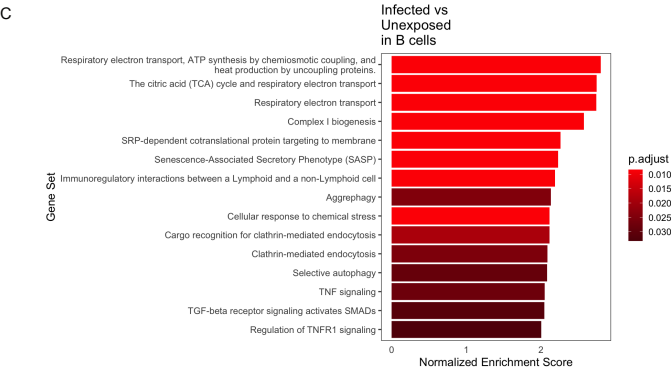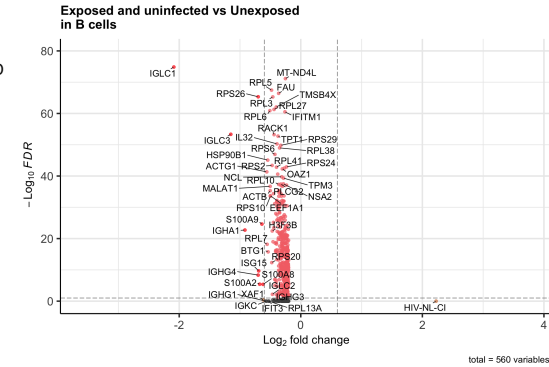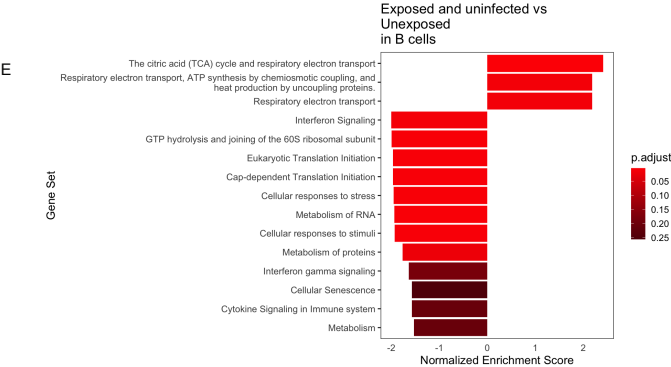

Supplement: Supplementary Figure 6 — Differential expression by HIV-1NL-CI infection and exposure in B cells. Violin plots of various B cell marker gene expression are shown in B cells (A). Volcano plots of genes differentially expressed in infected cells vs. unexposed cells (B) and exposed uninfected cells vs. unexposed cells (D). Colors denote significance and fold change cutoffs: red = absolute log2(fold change) ≥ 0.6 and adjusted p-value < 0.05, pink = absolute log2(fold change) < 0.6 and adjusted p-value <0.05, orange = absolute log2(fold change) ≥ 0.6 and adjusted p-value >0.05, grey = absolute log2(fold change) < 0.6 and adjusted p-value >0.05 (B, D). Bar plots illustrate the top 15 Reactome database gene sets enriched in infected cells vs. unexposed cells (C) and exposed uninfected cells vs. unexposed cells (E). Gene sets with adjusted p-value <0.05 were considered significant. A positive Normalized Enrichment Score (NES) value indicates enrichment (C, E). [file Image_6.pdf]

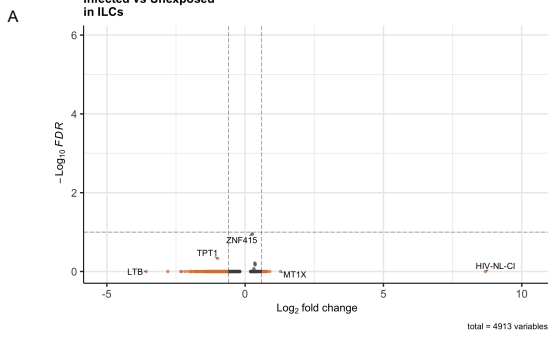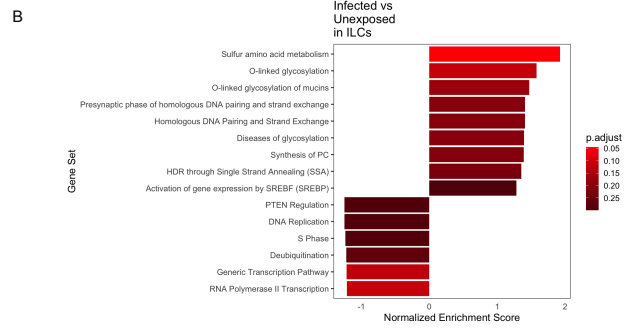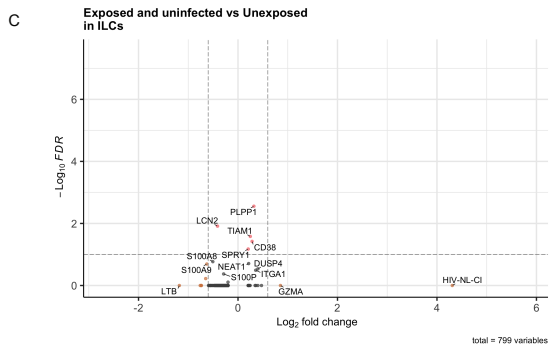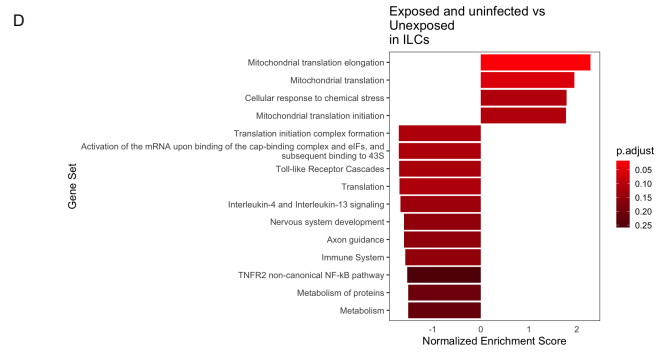

Supplement: Supplementary Figure 7 — Differential expression by HIV-1NL-CI infection and exposure in innate lymphoid cells (ILC). Volcano plots of genes differentially expressed in infected cells vs. unexposed cells (A) and in exposed uninfected cells vs. unexposed cells (C). Colors denote significance and fold change cutoffs: red = absolute log2(fold change) ≥ 0.6 and adjusted p-value < 0.05, pink = absolute log2(fold change) < 0.6 and adjusted p-value <0.05, orange = absolute log2(fold change) ≥ 0.6 and adjusted p-value >0.05, grey = absolute log2(fold change) < 0.6 and adjusted p-value >0.05 (A, C). Bar plots illustrate the top 15 Reactome database gene sets enriched in infected cells vs. unexposed cells (B) and exposed uninfected cells vs. unexposed cells (D). Gene sets with adjusted p-value <0.05 were considered significant. A positive Normalized Enrichment Score (NES) value indicates enrichment (B, D). [file Image_7.pdf]

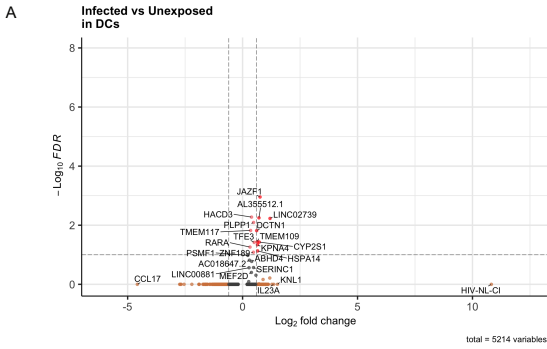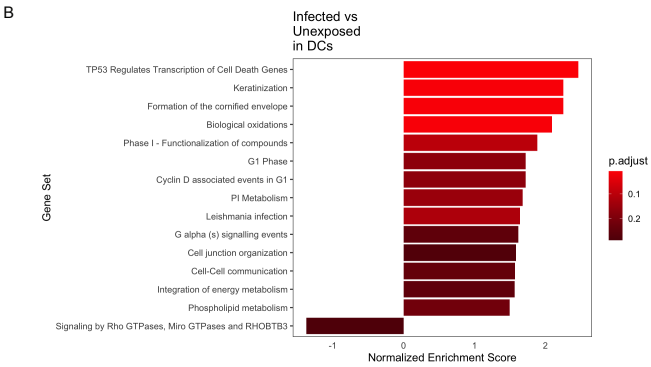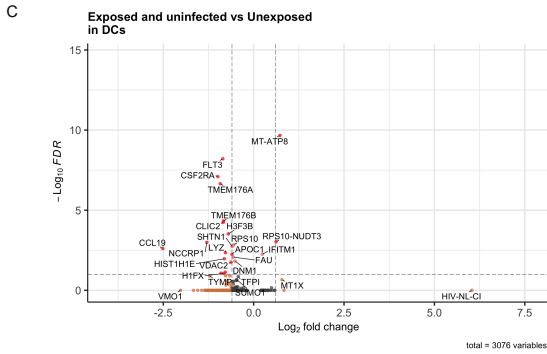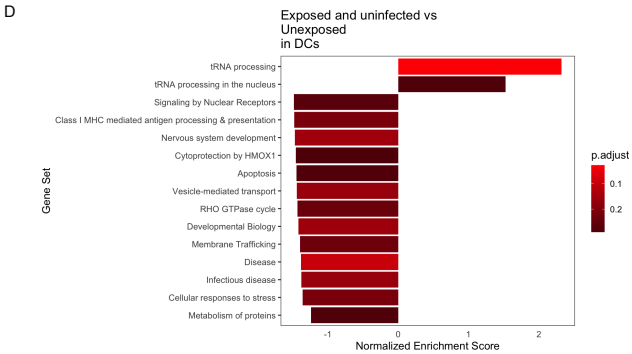

Supplement: Supplementary Figure 8 — Differential expression by HIV-1NL-CI infection and exposure in dendritic cells. Volcano plots of genes differentially expressed in infected cells vs. unexposed cells (A) and in exposed uninfected cells vs. unexposed cells (C). Colors denote significance and fold change cutoffs: red = absolute log2(fold change) ≥ 0.6 and adjusted p-value < 0.05, pink = absolute log2(fold change) < 0.6 and adjusted p-value <0.05, orange = absolute log2(fold change) ≥ 0.6 and adjusted p-value >0.05, grey = absolute log2(fold change) < 0.6 and adjusted p-value >0.05 (A, C). Bar plots illustrate the top 15 Reactome database gene sets enriched in infected cells vs. unexposed cells (B) and exposed uninfected cells vs. unexposed cells (D). Gene sets with adjusted p-value <0.05 were considered significant. A positive Normalized Enrichment Score (NES) value indicates enrichment (B, D). [file Image_8.pdf]

A

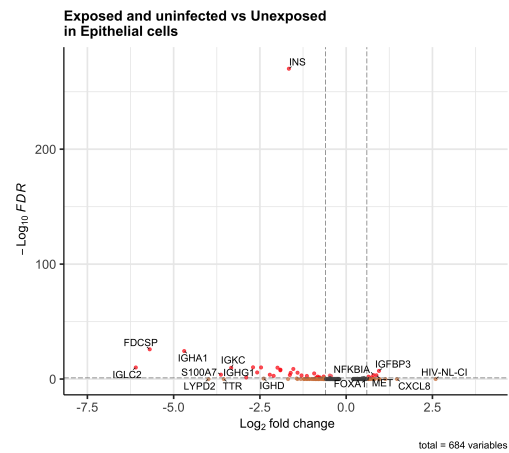

B

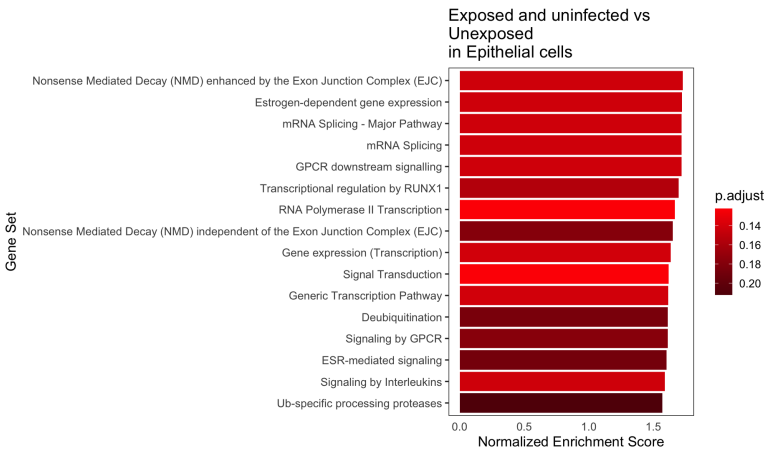

Supplement: Supplementary Figure 9 — Differential expression by HIV-1NL-CI infection and exposure in epithelial cells. Volcano plots of genes differentially expressed in exposed uninfected cells vs. unexposed cells. Colors denote significance and fold change cutoffs: red = absolute log2(fold change) ≥ 0.6 and adjusted p-value < 0.05, pink = absolute log2(fold change) < 0.6 and adjusted p-value <0.05, orange = absolute log2(fold change) ≥ 0.6 and adjusted p-value >0.05, grey = absolute log2(fold change) < 0.6 and adjusted p-value >0.05. Bar plots illustrate the top 15 Reactome database gene sets enriched in infected cells vs. unexposed cells (C) and exposed uninfected cells vs. unexposed cells (D). Gene sets with adjusted p-value <0.05 were considered significant. A positive Normalized Enrichment Score (NES) value indicates enrichment. [file Image_9.pdf]

## Complex I biogenesis

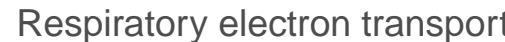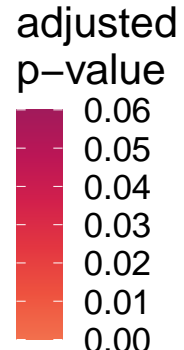

Supplement: Supplementary Figure 10 — Inflammasome gene expression by cell type. Dot plots illustrating enrichment of key oxidative phosphorylation and inflammasome signaling gene sets by cell type in infected cells vs. unexposed cells (left) and in exposed uninfected cells vs. unexposed cells (right). Dot color and size are proportional to the adjusted enrichment p-value. [file Image_10.pdf]
