# Supplementary material for: HIV-1 activates oxidative phosphorylation in infected CD4 T cells in a human tonsil explant model
Source: Front Immunol. 2023 May 30;14:1172938. doi: 10.3389/fimmu.2023.1172938 (PMC10266353; doi:10.3389/fimmu.2023.1172938)
Supplement: Supplementary Figure 4 — Patterns of T cell expression with HIV-1 infection and exposure. Volcano plot of genes differentially expressed (A), and bar plot illustrating the top 15 Reactome database gene sets enriched in exposed uninfected T cells vs. exposed T cells (B). Colors denote significance and fold change cutoffs: red = absolute log2(fold change) ≥ 0.6 and adjusted p-value < 0.05, pink = absolute log2(fold change) < 0.6 and adjusted p-value <0.05, orange = absolute log2(fold change) ≥ 0.6 and adjusted p-value >0.05, grey = absolute log2(fold change) < 0.6 and adjusted p-value >0.05. Gene sets with adjusted p-value <0.05 were considered significant (A). A positive Normalized Enrichment Score (NES) value indicates enrichment (B). Dot plots demonstrate the expression of 5 oxidative phosphorylation genes in each sub-cell type, comparing unexposed, exposed uninfected, and infected cells. Dot size is proportional to the percentage of cells within a group. The dot color indicates the average expression across the group (red = high, blue = low) (C). [file Image_4.pdf]

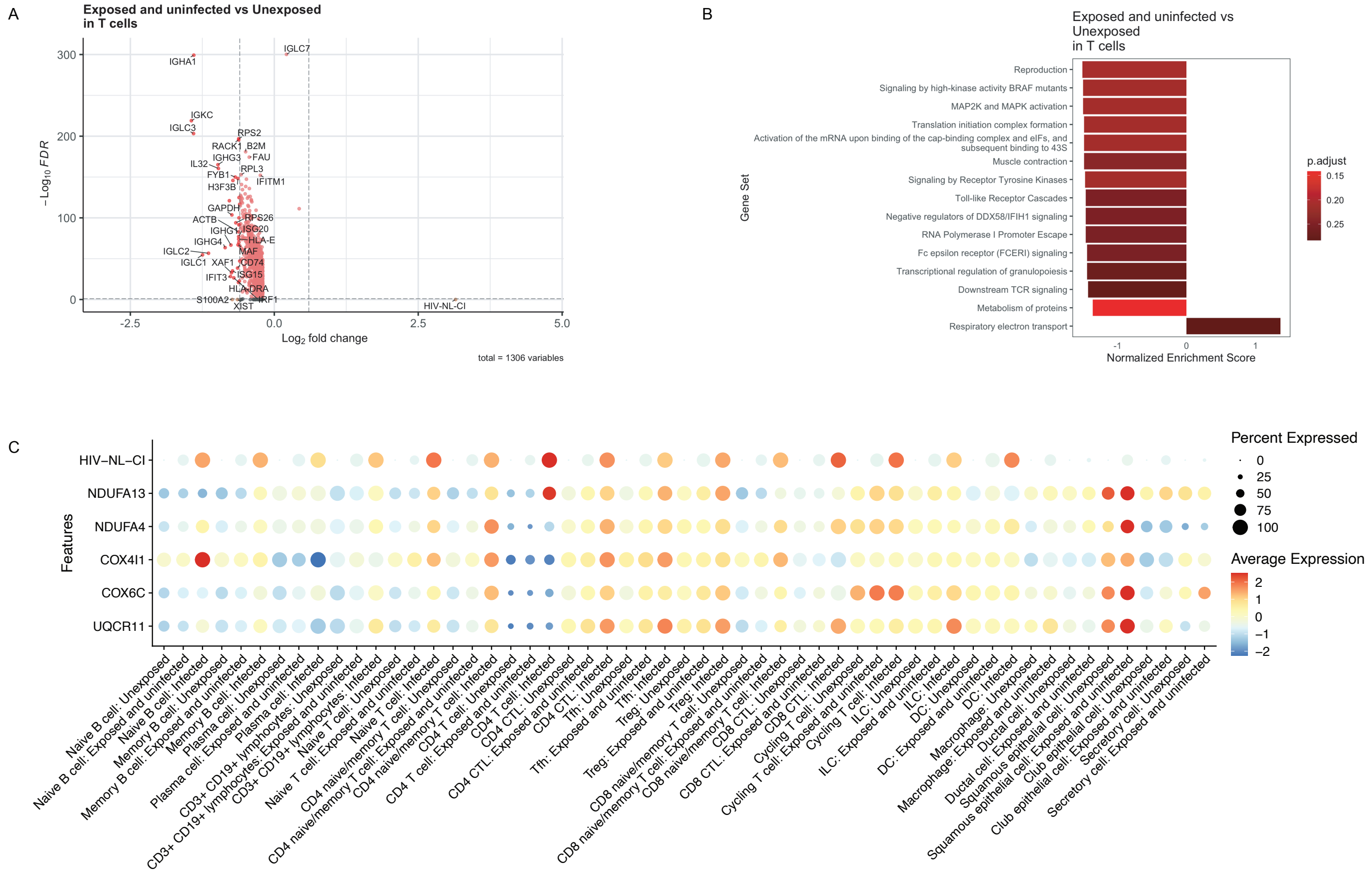
A
Exposed and uninfected vs Unexposed in T cells
IGLC7
IGHA1
IGKC
IGLC3
RPS2
RACK1
B2M
IGHG3
FAU
IL32
RPL3
FYB1
IFITM1
H3F3B
GAPDH
ACTB
RPS26
IGHG1
ISG20
IGHG4
HLA-E
IGLC2
MAF
IGLC1
XAF1
CD74
IFIT3
ISG15
HLA-DRA
S100A2
IRF1
XIST
HIV-NL-CI
Log2 fold change
−Log10 FDR
total = 1306 variables
B
Exposed and uninfected vs Unexposed in T cells
Reproduction
Signaling by high-kinase activity BRAF mutants
MAP2K and MAPK activation
Translation initiation complex formation
Activation of the mRNA upon binding of the cap-binding complex and eIFs, and subsequent binding to 43S
Muscle contraction
Signaling by Receptor Tyrosine Kinases
Toll-like Receptor Cascades
Negative regulators of DDX58/IFIH1 signaling
RNA Polymerase I Promoter Escape
Fc epsilon receptor (FCERI) signaling
Transcriptional regulation of granulopoiesis
Downstream TCR signaling
Metabolism of proteins
Respiratory electron transport
Gene Set
Normalized Enrichment Score
p.adjust
C
HIV−NL−CI
NDUFA13
NDUFA4
COX4I1
COX6C
UQCR11
Features
Percent Expressed
Average Expression
